# An augmentation aided concise CNN based architecture for COVID-19 diagnosis in real time

**DOI:** 10.1038/s41598-024-51317-y

**Published:** 2024-01-11

**Authors:** Balraj Preet Kaur, Harpreet Singh, Rahul Hans, Sanjeev Kumar Sharma, Chetna Kaushal, Md. Mehedi Hassan, Mohd Asif Shah

**Affiliations:** 1 Department of Computer Science and Engineering, DAV University, Jalandhar, India; 2 Department of Computer Science and Engineering, Thapar Institute of Engineering and Technology, Patiala, India; 3 Department of Computer Science and Applications, DAV University, Jalandhar, India; 4 Chitkara University Institute of Engineering and Technology, Chitkara University, Punjab, 140401 India; 5 Computer Science and Engineering Discipline, Khulna University, Khulna, 9208 Bangladesh; 6 Department of Economics, Kebri Dehar University, Kebri Dehar, 250, Ethiopia; 7 Centre of Research Impact and Outcome, Chitkara University Institute of Engineering and Technology, Chitkara University, Rajpura, 140401, Punjab India; 8 Division of Research and Development, Lovely Professional University, Phagwara, 144001, Punjab India

**Keywords:** Computational biology and bioinformatics, Medical research

## Abstract

Over 6.5 million people around the world have lost their lives due to the highly contagious COVID 19 virus. The virus increases the danger of fatal health effects by damaging the lungs severely. The only method to reduce mortality and contain the spread of this disease is by promptly detecting it. Recently, deep learning has become one of the most prominent approaches to CAD, helping surgeons make more informed decisions. But deep learning models are computation hungry and devices with TPUs and GPUs are needed to run these models. The current focus of machine learning research is on developing models that can be deployed on mobile and edge devices. To this end, this research aims to develop a concise convolutional neural network-based computer-aided diagnostic system for detecting the COVID 19 virus in X-ray images, which may be deployed on devices with limited processing resources, such as mobile phones and tablets. The proposed architecture aspires to use the image enhancement in first phase and data augmentation in the second phase for image pre-processing, additionally hyperparameters are also optimized to obtain the optimal parameter settings in the third phase that provide the best results. The experimental analysis has provided empirical evidence of the impact of image enhancement, data augmentation, and hyperparameter tuning on the proposed convolutional neural network model, which increased accuracy from 94 to 98%. Results from the evaluation show that the suggested method gives an accuracy of 98%, which is better than popular transfer learning models like Xception, Resnet50, and Inception.

## Introduction

Coronavirus^1^ was identified in Wuhan, China, in 2019, and it has affected more than 760 million people around the globe^2^. The virus causes respiratory diseases such as Middle East Respiratory Syndrome, Severe Acute Respiratory Syndrome (SARS)^3^, and other deadly complications. The most common symptoms are cough, sore throat, headache, fever and fatigue (https://covid19.who.int/). The virus is passed from person to person by droplets of breath. During past COVID 19 waves, the sudden surge in cases made it difficult for the laboratories to confirm positive or negative cases using RT-PCR as it is a time-consuming method and has high false-negative rates^4^, and is costly also. Therefore, development of real time diagnostic tools, which can be executed in mobile and edge devices is the need of the hour^5^. Since most diagnostic centers already have X-ray machines, and because acquiring an X-ray takes less time than getting the RT-PCR done, using chest X-rays of patients^6^ satisfies the urgent need for a speedy diagnostic approach.

**Figure 1:**
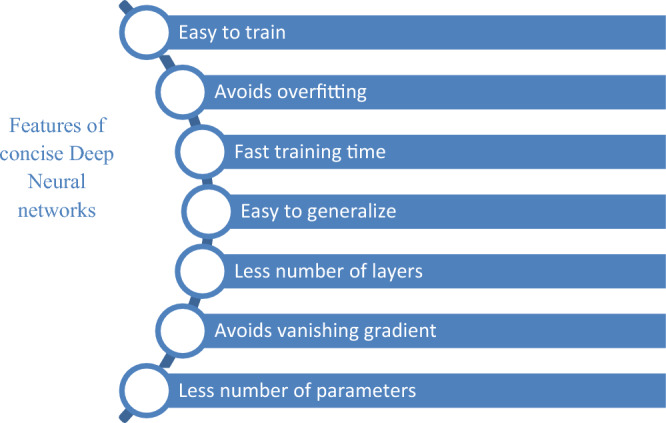
Features of concise CNNs.

Deep learning^7,8^ is one of the most promising techniques that provides efficient results in the accurate diagnosis of the diseases from images and is widely used in the medical field to diagnose severe diseases at early stages^9^. It is made up of input layer, activation functions, hidden layers and also output layer. The mathematical equation in each step with feed forward and backward functions can help in finding better results^10^. An activation function is used to activate and deactivate the neurons and basically defines the output of a node. Convolutional neural networks (CNN)^11,12^ are deep learning neural network made up of neurons which are experienced, self-optimized and are used primarily by researchers working in the field of disease diagnosis from images. CNN’s key popularity is attributed to its ability to automatically learn functions from domain-specific images^13^. Furthermore, transfer learning models^14^ saves knowledge from one problem and can apply that knowledge on another problem. But the conventional CNN models such as Resnet50, AlexNet, Inception and Xception, etc., cannot be run on low computing power devices such as tablets, embedded chips, mobile phones and hence cannot be given to real time applications^15,16^. These conventional models are also complex, need a lot of training time. To overcome these shortcomings, the lightweight^17^ and concise models of CNNs are being developed which are having lesser number of parameters than the conventional CNNs so that they can be executed on devices with low computing power and smaller memory requirements. Figure 1 shows the features of concise CNNs.

In the case of CNNs, there is also a need to preprocess the image to get a better classification and for that purpose, image enhancement techniques^18^ are used in which mask and filters upgrade the quality of image. In addition to that data augmentation techniques^19^ increase the training data to upgrade the successful rate of model. The goal of this study is to present a new, simple CNN-based model for diagnosing COVID 19 from X-ray pictures, and the proposed model has been compared to existing transfer learning methods using a number of different criteria. The following are the primary contributions of this research work: (1) a novel set of layers, as well as image enhancement and hyperparameter tuning of parameters, have been suggested for the classification of COVID-19, normal, and pneumonia cases. (2) In order to prevent the models from overfitting, data augmentation has also been carried out. (3) The proposed framework may be used as one of the effective methods for classifying data in the medical industry. Furthermore, it helps radiologists diagnose and treat ailments earlier.

The paper is divided into the sections listed below. The second section discusses the literature review and the third section explains our suggested model. Materials and methods are discussed in fourth section. Fifth section includes a description of the experimental outcomes. Conclusions and future work are discussed in sixth section.

## Literature survey

Recently, most frequently research has been going on in the domain of diagnosis disease using CNN from images. This section summarizes some of the existing works for disease diagnosis. The comparison of models performances are shown in Table 1.

**Table 1:** State of the art techniques.

Author's (year)	Disease	Image type	Models used	Accuracy (%)
Anubhav Sharma et al. (2022)^20^	COVID-19	Chest X-ray	CNN Mobile Net, VGG16, Resnet50, Xception	96.4
Khalid EL Asnaoui et al. (2021)^21^	COVID-19	Chest X-ray and CT dataset	CNN Mobile Net, VGG16, Resnet50, Xception	96
SadmanSakib et al. (2020)^22^	COVID-19	Chest X-ray	CNN, GAN, LSTM	93
MortezaHeidari et al. (2020)^19^	COVID-19	Chest X-ray	CNN, VGG16, VGG19, Xception, Inception, DenseNet121	94.5
Aras M. Ismael et al. (2021)^23^	COVID-19	Chest X-ray	CNN, SVM	94.7
Naveena et al. (2022)^24^	Diabetes	PIMA and UCI	CNN, RNN and Crow search algorithm	96
Aggarwal et al. (2021)^25^	COVID-19	Chest X-ray	MobileNetV2, ResNet50, InceptionV3, NASNetMobile, VGG16, Xception, InceptionResNetV2 DenseNet121	97
Dipayan Das et al. (2020)^26^	COVID-19	Chest X-ray	Truncated Inception Net, Inception Net V3	99.6
JieHou et al. (2021)^27^	COVID-19	Chest X-ray	Deep convolution neural network (DCNN)	96
Azher Uddin et al. (2021)^21^	COVID-19	Chest X-ray	VGG16, InceptionV3, MobileNetV2, and ResNet50	98
Shashwat Sanket et al. (2020)^28^	COVID-19	Chest X-ray	VGG16, InceptionV3, VGG19, and ResNet50	98.4

Litjens et al.^29^ proposed application aspects in deep learning. The different deep learning techniques extracted the spatial features from sophisticated image data i.e. CT, X-ray images, color Fundus images, ultrasound image and implemented models, which can be helpful in hospitals to detect severe diseases such as diabetic retinopathy, skin lesion, bone fracture and breast cancer at their early stages. Kermany et al.^30^ used optical coherence tomography images dataset to detect viral pneumonia and macular degeneration and diabetic retinopathy. Cao et al.^3^ introduced deep learning, and the image analysis is done by deep learning architecture such as RNN, CNN and Stacked machine auto encoder. With these models, the detection of pediatric pneumonia with chest X-ray images can be done. The authors also presented the challenges in handling unlabeled data, privacy issues in the medical field, and many more. Jaiswal et al.^31^ in their work used the region of interest, align convolution layer and pixel-wise segmentation of disease.

Toğaçar et al.^14^ proposed a minimum redundancy maximum relevance (mRMR) model for the diagnosis of pneumonia. The three knowledge transfer models, namely, VGG-16, Alex Net, and VGG-19,are used in the proposed architecture. Moreover, decision tree, linear Discriminant analysis, k-nearest neighbor, and support vector machines are used for grouping using features generated by transfer learning model. Singh et al.^32^ proposed multi-objective differential evolution model for the classification of the COVID 19 disease. An exponential crossover algorithm is used. The proposed model gives high accuracy as compared to adaptive neuron fuzzy inference system, artificial neural network, and CNN types.

Das et al.^33^ designed an Xception model to diagnosed COVID infection using chest X-ray dataset containing three classes pneumonia and COVID 19 negative, COVID 19 positive, and other infections except for COVID. The features are extracted by using different masks applied to the convolution layer. As a loss function, the cross-entropy is utilized. Brunese et al.^34^ built two models: the first model assesses whether the picture belongs to a healthy patient or a patient suffering from general pulmonary illness. If the patient has a general pulmonary condition, the X-ray picture is sent to the second model, which checks whether it is a COVID 19 patient or pulmonary disease only.

Liu et al.^7^ suggested a model for dental disease diagnosis utilizing a mask region-based convolution neural network with classification of seven different dental diseases. The model uses an IoT platform for patients to upload their dental images. A broad-level prototype is also given in the paper for dental image acquisition. Jain et al.^35^ presented four phases of model where ResNet50 network is used to differentiate between bacterial pneumonia and pneumonia. Varela et al.^36^ suggested approach uses feature extraction to minimize the number of pixels, grey level co-occurrence matrix features that focus on the spatial relationship between pixels, and the local binary patterns method to encode the pixel values. Marques et al.^37^ has been suggested efficientnetb4 model is a convolution neural network. Ezzat et al.^38^ suggested a technique to identify the optimal settings for hyperparameters, the gravitational search method is utilized as an optimization tool. The new method is contrasted with Social Ski Driver-Dennsenet121. Data preparation, hyperparameter selection, and the learning step for COVID 19 diagnosis are all part of the technique. Hassantabar et al.^39^ has been proposed technique for detecting COVID 19 patients. Two approaches are utilized for diagnosis. The first is a deep neural network, while the second is an image segmentation approach for detecting diseased areas. Table 1 summarizes studies relating to COVID 19 and CNN architecture on chest x ray images (Type1) and PIMA and UCI (Type2), as well as information on additional approaches utilized in the papers.

Various studies have explored the application of deep learning techniques across diverse imaging modalities, including CT scans, X-rays, color fundus images, ultrasound, and optical coherence tomography^40–42^. The investigated diseases range from diabetic retinopathy and skin lesions to bone fractures, breast cancer, viral pneumonia, and COVID-19^13,43^. These studies employ a variety of deep learning architectures such as RNNs, CNNs, and stacked machine auto encoders. Notably, researchers have addressed challenges like handling unlabeled data and privacy issues in the medical field^44^. Key findings include the efficacy of models like Xception for diagnosing COVID-19 from chest X-ray images, the use of multi-objective models for COVID-19 classification, and innovative approaches like dental disease diagnosis using a mask region-based CNN^45^. The comparison in Table 1 underscores the performance of different models in COVID-19 diagnosis and CNN architecture across chest X-ray images and datasets like PIMA and UCI. Overall, these studies demonstrate the versatility and potential impact of deep learning in advancing early disease detection and diagnostic accuracy in medical imaging.

## Proposed concise CNN based architecture

**Figure 2:**
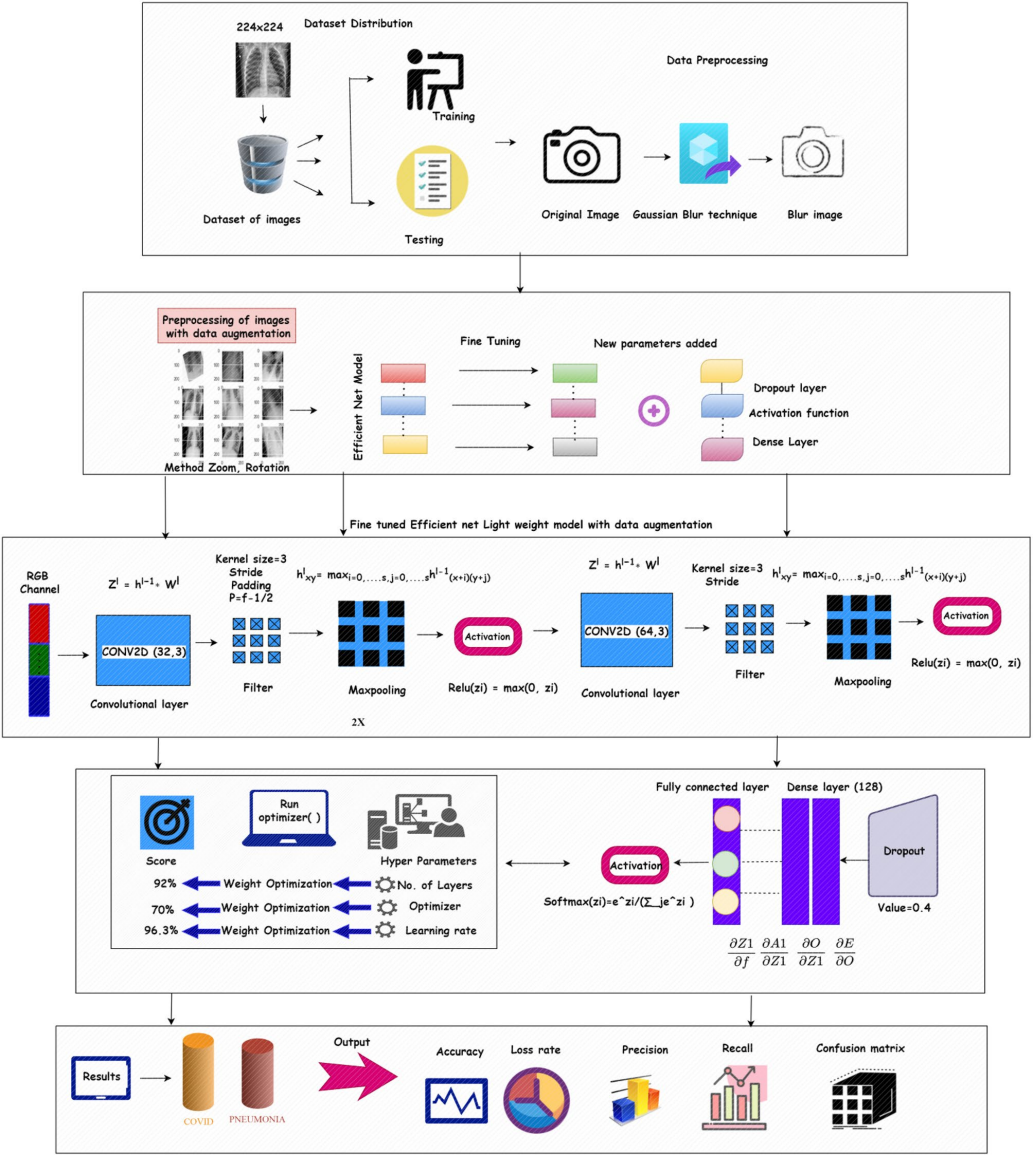
Architecture of proposed model (source of clipart images: draw.io).

The framework of a convolutional neural network depends on the number of layers, activation function, optimizer, number of filters and batch size^46–48^. Figure 2 represents the proposed architecture of the COVID 19 diagnosis structure. The proposed model has been derived from the baseline Efficient Net model^46^. In contrast to the more complex architectures, the goal is to develop a concise CNN model that can identify picture modification^40^. The layers of the efficient Net model have are modified by replacing the MBconv layer with a Conv2D layer and also by updating the values of filter in layers additionally,the dropout layer is added with a 0.4 value to reduce overfitting of the model and add regularization. The proposed architecture is a sequential model. Additionally, the layers are added in the sequence order to build the CNN architecture. The proposed CNN model contains Conv2D, Maxpooling2D, Dropout, the Relu activation function, dense/fully connected layer. The suggested model has nine total layers: three convolutional, three maxpooling, three Relu, two dense, one dropout, and a fully connected layer.

- The size of image as input is 224 × 224 × 3, i.e., 224 is height and width of image and 3 is image channel value as RGB. The first convolution layer (L1) represent the first layer of model takes an input of size 224 × 224 × 3 and has kernel size 3 × 3 which produces 32 features maps as result.
- The second convolution layer (L2) has 32 filters and has kernel size 3 × 3 which produces 32 features maps as result.
- The third convolution layer (L3) contain 64 filters with kernel size 3 × 3 which produces tensor of 64 features maps as result.
- To tackle the overfitting problem, the above layers are followed by dropout layer with 64 filters having kernel size of 3 × 3. Dropout layer is followed by flattening layer. The flatten layer converts the data into 1-D form.
- In last the dense/fully connected layer is added with 128 filters and the efficiency of the model is improved by Relu as activation function which produces 258 features. This layer produces the output.

Convolutional layers are used in conjunction with the most common Rectified linear unit (ReLU) activation function to increase the performance and generalization by introducing non-linearities to the network. The vanishing gradient issue that may be seen in other forms of activation functions is eliminated by ReLU by correcting the values of inputs less than zero. ReLU's key benefit is quicker execution, which shortens computation time. The maxpool2D is used with each convolution layer to extract the best features. The description of each layer is represented in Table 2 in which Conv2D as T1 layer, Max_pooling2D as T2 layer, Dropout as T3 layer, Flatten as T4 layer and Fully connected as T5 layer.

**Table 2:** Layer architecture of proposed model.

Layer no	Operation	No. of filter	Kernel	No. of parameter
1	Conv2D	32	3 × 3	896
2	Max_Pooling2D	32	1 × 1	N/A
3	Conv2D	32	3 × 3	9248
4	Max_Pooling2D	32	3 × 3	N/A
5	Conv2D	64	3 × 3	18,496
6	Max_Pooling2D	64	1 × 1	N/A
7	Dropout	64	3 × 3	N/A
8	Flatten	50,176	1 × 1	N/A
9	Fully connected layer	128	3 × 3	6,422,656

The filter applied on the image is represented in Eq. (1). The h is a kernel and input image is represented as f. The resulting matrix of indexes of rows and columns is marked as q, r. ∑ a sign is used to add all values with limits j and k.

1 $$R[q,r] =[f*h][q,r]= {\sum }_{j}\sum_{k}h\left[j,k\right] f[q-j,r-k]$$

After this process, the filter is placed over the image and the value is multiplied by the value from the image. Then all values sum up and the feature map is generated. The padding is added to the image to fix the size in proper form. Equation (2) is used for padding.

2 $$p=\frac{ f-1}{2}$$

The preprocessing part with CONV2D now moves to the pooling process. The formula for the pooling function is defined in Eq. (3). In the pooling process, we find the maximum and average according to the pool type. It is a technique to get sample feature maps from all features. It extracts features that contain high value during the sliding window extraction process. Here, s is the stride, n_H_ is a size of height, nc is number of channels, n_w_ is size of width.

3 $$\mathrm{Pooling }= (\left[ \frac{{n}_{H}+2p-f}{s}+1\right],\left[ \frac{{n}_{w}+2p-f}{s}+1\right],nc, ):s>0,$$

For improved results, Relu is employed as an activation function in each CONV2D layer and maxpooling layer. The function work as Eq. (4), where x is the input value

4 $$Relu(x) = max(0,x)$$

The convolution layer with maxpooling value is then direct to the feed forward function to calculate the value for the next step, Eq. (5) represents the functioning of this process

5 $$Z[I] = W[I] \cdot AF[I-1] + b[I]$$

6 $$AF[I] = g[I] (Z[I])$$

Here, g is the activation function in Eq. (6), firstly the value of *Z* is calculated from the previous layer with *W* tensor and then bias *b* is added to it.

After calculating it, we move to the calculation of derivatives which will be used to update the value of parameter also known as gradient descent. The formula of a partial derivative as

7 $$dAF\left[I\right]= \frac{\partial L}{\partial A\left[I\right]} dZ\left[I\right]= \frac{\partial L}{\partial Z\left[I\right]} dW\left[I\right]= \frac{\partial L}{\partial W\left[I\right]} db\left[I\right]= \frac{\partial L}{\partial b\left[I\right]}$$

dW and db are parameters that work on the present layer. According to the chain rule, the result is Eq. (8)

8 $$dZ[I] = dAF[I] * g{\prime}(Z[I] )$$

After the backpropagation process, hyperparameters tuning is the next step which includes checking parameter values with different patterns based on the performance of the model. The parameter used for tuning are: loss function, learning rate, optimizer and number of neurons.

- Loss function: It is used to compute the model error. The gradients may be calculated from the loss function and used to update the weights. To generate output, the suggested model uses a sparse categorical cross entropy loss function and the mathematical operation of which is shown in Eq. (9)
  9 $$L= \sum_{j=1}^{M}yi log(\widehat{y}i)$$
  where y hat represent the outcome produced by the model and y represents the expected outcome.

(b) Optimizer: The goal of an optimizer is to minimize losses by adjusting relevant model parameters like the learning rate and the weights. In the proposed approach, RMSprop is used as optimizer. The RMSprop takes the cumulative sum of the squared gradient represented in Eqs. (10) and (11)
  10 $$wt+1 = wt- \frac{\alpha t }{(vt + e)1/2}* \left[\frac{\delta l}{\delta wt}\right]$$
  11 $$vt=\beta vt-1 + (1-\beta )*\left[\frac{\delta l}{\delta wt}\right]^{2}.$$

Here, $\alpha t$ learning rate at time *t,* $\delta wt$ derivative of weight at time t and $\delta l$ derivative of the loss function, v_t_ sum of the square of past gradient, ${\text{e}}$ small positive constant (10^–8^) and β is moving average parameter (constant, 0.9). Dense Layer receives input from all neurons of the previous layer along with the Relu activation function. The dense layer return output is represented in Eq. (12)

12 $$o = g(dot(I, K)+b)$$

**Figure 3:**
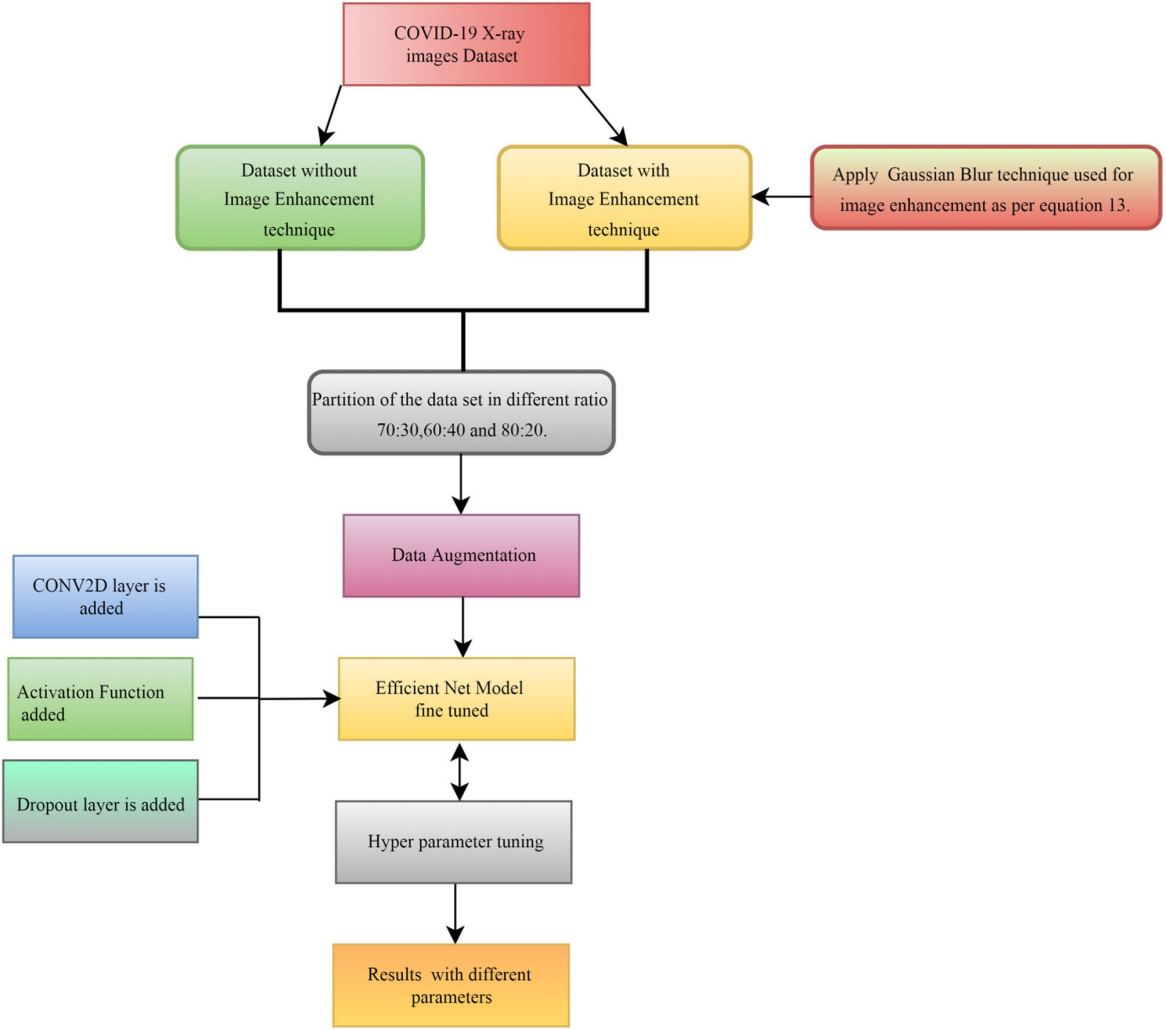
Phases undertaken in the proposed framework.

In the above equation, *o* is output *g* is the activation function, dot represents numpy function for calculation, *I* is input. K represents the weight data, while b is the bias used to optimize the model. Figure 3 depicts the study's step-by-step process. The classification process of CNNs is to process input images through convolutional, activation, pooling, and fully connected layers. Training involves optimizing weights via backpropagation to minimize a loss function. The trained model predicts image classes by analyzing learned features.

## Materials and methods

The description of the dataset^49^ that was utilized in the experiment is configured into two types. The firstly used dataset in the experiment is dataset with data augmentation^44^. The second dataset used in the experiment is with image enhancement using hyper parameter tuning, data augmentation and Gaussian Blur.

### Dataset description

**Figure 4:**
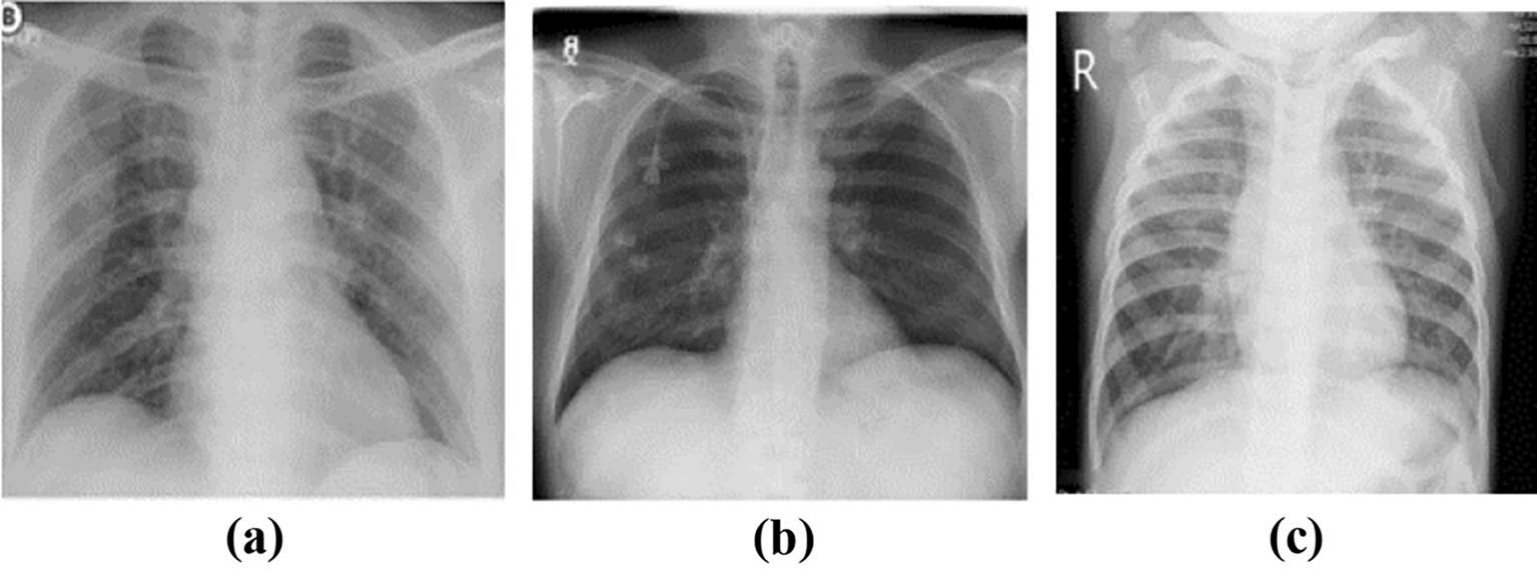
Sample image from each class (**a**) COVID 19 infected, (**b**) normal and (**c**) pneumonia.

The suggested model for detecting COVID 19 illness was evaluated using a dataset of publically accessible conventional chest X ray images^50^. The collection includes 3616 COVID 19 positive cases, 10,192 Normal pictures, 6012 Lung Opacity, and 1345 viral Pneumonia images. Only two of the four types of images presented were taken into account in our experiments, i.e., COVID 19 positive and viral Pneumonia. Every image is a grayscale image consisting of $299\times 299$ pixels. Figure 4 shows the sample image from each class of the test dataset. Total of 1000 images are taken from a dataset and then divided into different three samples.

### Image enhancement

Gaussian blur feature is derived by blurring an image using Gaussian function. This technique upgrade the quality of an image and is helpful in finding inadequate information for image interpretation^51^. The spatial filtering, slicing, stretch, edge sharpening and other methods are used in this technique. The method reduces noise and increases smoothening of image. The process is achieved by convolving on image with Gaussian kernel. The formula used for the process is shown in Eq. (13)^18^:

13 $${G}_{2D}(x,y,{\sigma }^{2}) = \frac{1}{2\pi {\sigma }^{2}}{e}^{-}\frac{-{x}^{2}+{y}^{2}}{2{\sigma }^{2}}$$

**Figure 5:**
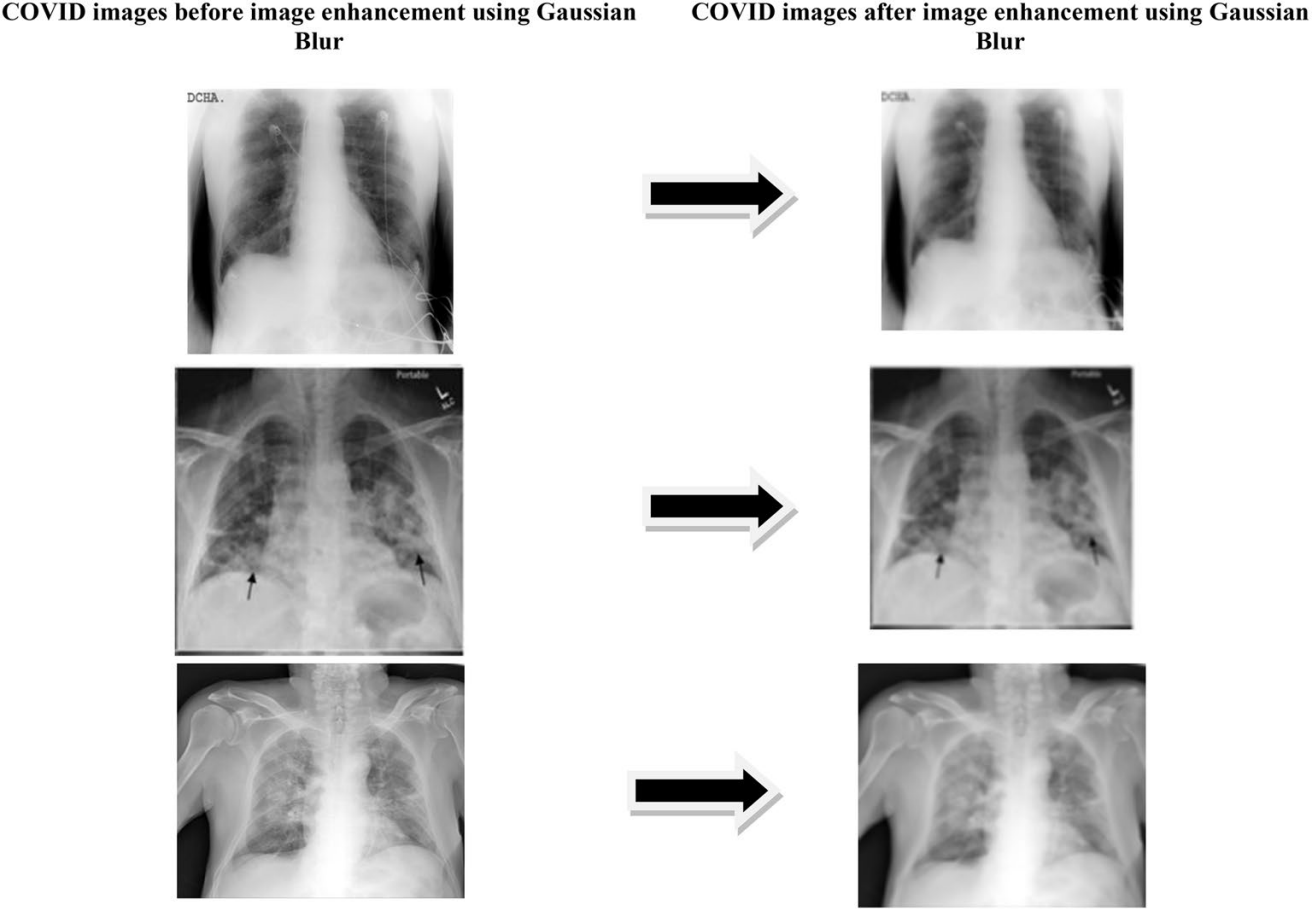
Image enhancement result on COVID dataset.

**Figure 6:**
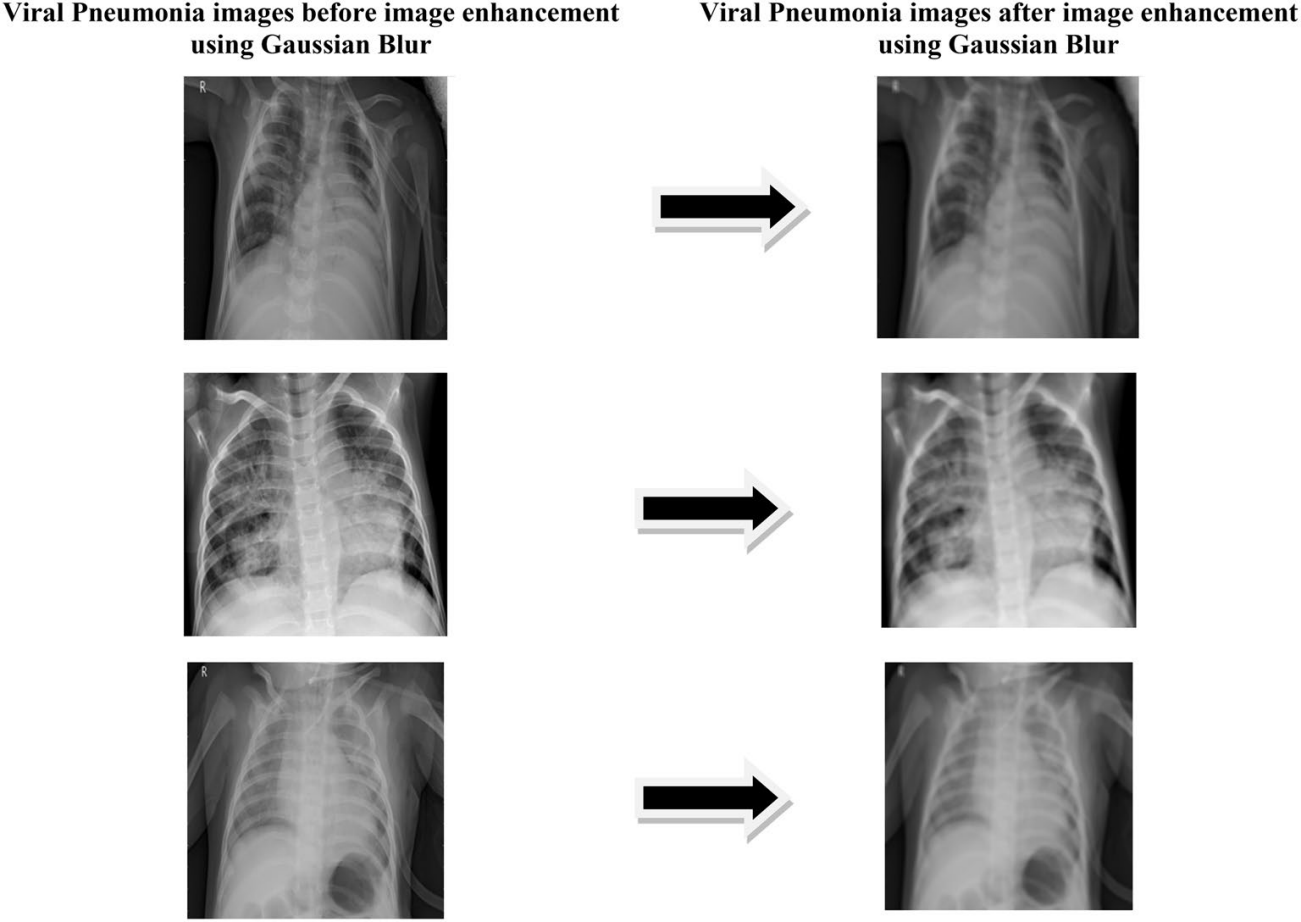
Image enhancement result on viral pneumonia dataset.

Here the distribution by standard deviation is denoted by *σ* and x, y are location indices. The Gaussian distribution mean value depends on the value of *σ* which influence the extent of blurring affect around pixel. The COVID and viral pneumonia images after and before Gaussian blur is shown in Figs. 5 and 6.

The opencv2 is used to implement Gaussian blur. The three functions are used as argument in the process i.e. img used to modified the image, sigma used in the x and y direction and truncate used to determining the limits of the approx. The Gaussian filter takes the x, y pixel and returns a single number by calculating the weighted average based on the normal distribution.

Figure 5 shows the images of chest X ray of covid class with and without image enhancement using Gaussian Blur^18^ technique. Figure 6 shows the images of chest X-ray of Viral-pneumonia classes with and without image enhancement using Gaussian Blur technique. The paper results contain experimentation on two type of dataset i.e. with Gaussian Blur images dataset and without Gaussian Blur images dataset.

#### Methodology of Gaussian Blur

**Figure:**
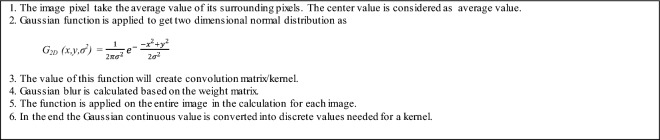
Algorithm of Gaussian Blur

The CLAHE^52^ and histogram equalization^53^ techniques are experimented before the selection of Gaussian Blur. The CLAHE define as contrast limiting adaptive histogram equalization which refine the images with high intensity. To improve contrast of image, histogram equalization is used. The other two techniques give less accuracy as compared to the blur technique^51^.

#### Data augmentation

**Figure 7:**
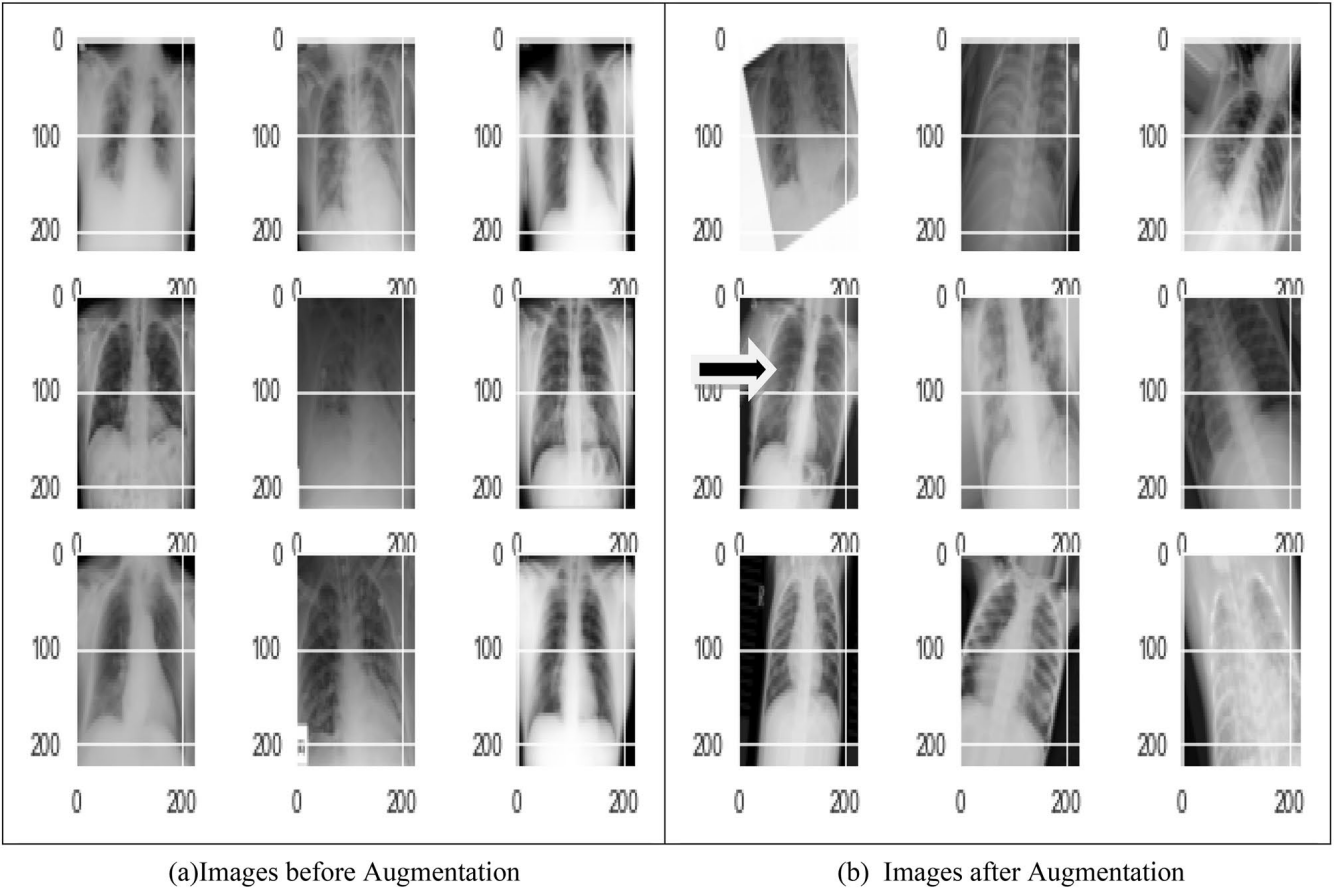
(**a**) Images before augmentation (**b**) images after augmentation.

In order to make training data more generalizable and applicable, data augmentation involves transforming images in various ways, such as rotating, flipping, and resizing^54^. Figure 7 shows the images without data augmentation and with data augmentation^19^. It increases the size of samples used as training set by applying different techniques written in the Table 2 which help the model to extract features and understand the image. The technique provide good results for enhancing the performance and is used to reduce over fitting^45^. The data augmentation methods is represent in Table 3 with different parameters.

**Table 3:** Data augmentation methods.

Augmentation method	Value
Rescale	1/255
Rotation range	30
Zoom range	0.2
Width shift range	0.1
Height shift range	0.1
Horizontal flip	True

The augmentation algorithms include kernel filters, geometric transformations, random erasing, mixing images, color space augmentations, etc. The above results show that preprocessing of image with data augmentation can increase the precision of classification and reduces the overfitting problem.

### Evaluation parameters

Based on the confusion matrix, we will determine the class-wise performance of our model based on the following performance metrics^55^.

1. True positive (TP): These are instances in which both the predictive and actual class are true(P).
2. True negative (TN): True negatives arise when both the expected and actual classes are false(N).
3. False negative (FN): These are instances in which the data item's real class is true (P), but the classification model wrongly labels it as false (N).
4. False positive (FP): These are instances when the data item's real class is false (N), but the classification model mistakenly labels it as true (P).
5. Accuracy: Accuracy is a percentage of correct predictions to total predictions. Equation (14) defines the accuracy formula:
  14 $$Accuracy=\frac{TP+TN}{TP+TN+FP+FN}$$
6. Loss: The difference between the predicted and actual value is the loss. It is a way for calculating loss. Equation (15) depicts the loss formula, where y represents the predicted outcome and y hat represents the model's output
  15 $$L= -(yi log(\widehat{y}i) +(1- yi) log(1-(\widehat{y}i))$$
7. Execution time: The time the model takes from start to finish of execution.
8. Recall: The percentage of total relevant results properly categorized by the model is referred to as recall.
  16 $$Recall = \frac{TP}{{TP + FN}}$$
9. Precision: It is the percentage of relevant results in your results. The formula is as follows:
  17 $$Precision = \frac{TP}{{TP + FP}}$$
10. F-Measure: The F-measure represents the harmonic mean of accuracy and recall. It is determined as follows:
  18 $$F{ - }Measure = \frac{2*Recall*Precision}{{Recall + Precision}}$$

## Experimental results and discussion

This part represents and analyze the results obtained after performing experiments in three different scenarios. All the proposed approaches have been executed with python using Tensor flow and Keras libraries^38^. For the analysis of results, dataset is categorized into different ratios as represented in Table 4.

**Table 4:** Dataset distribution.

	Scenario-1	Scenario-2	Scenario-3
Training	70	60	80
Testing	30	40	20

### Experimental results without image enhancement

#### Scenario 1

**Figure 8:**
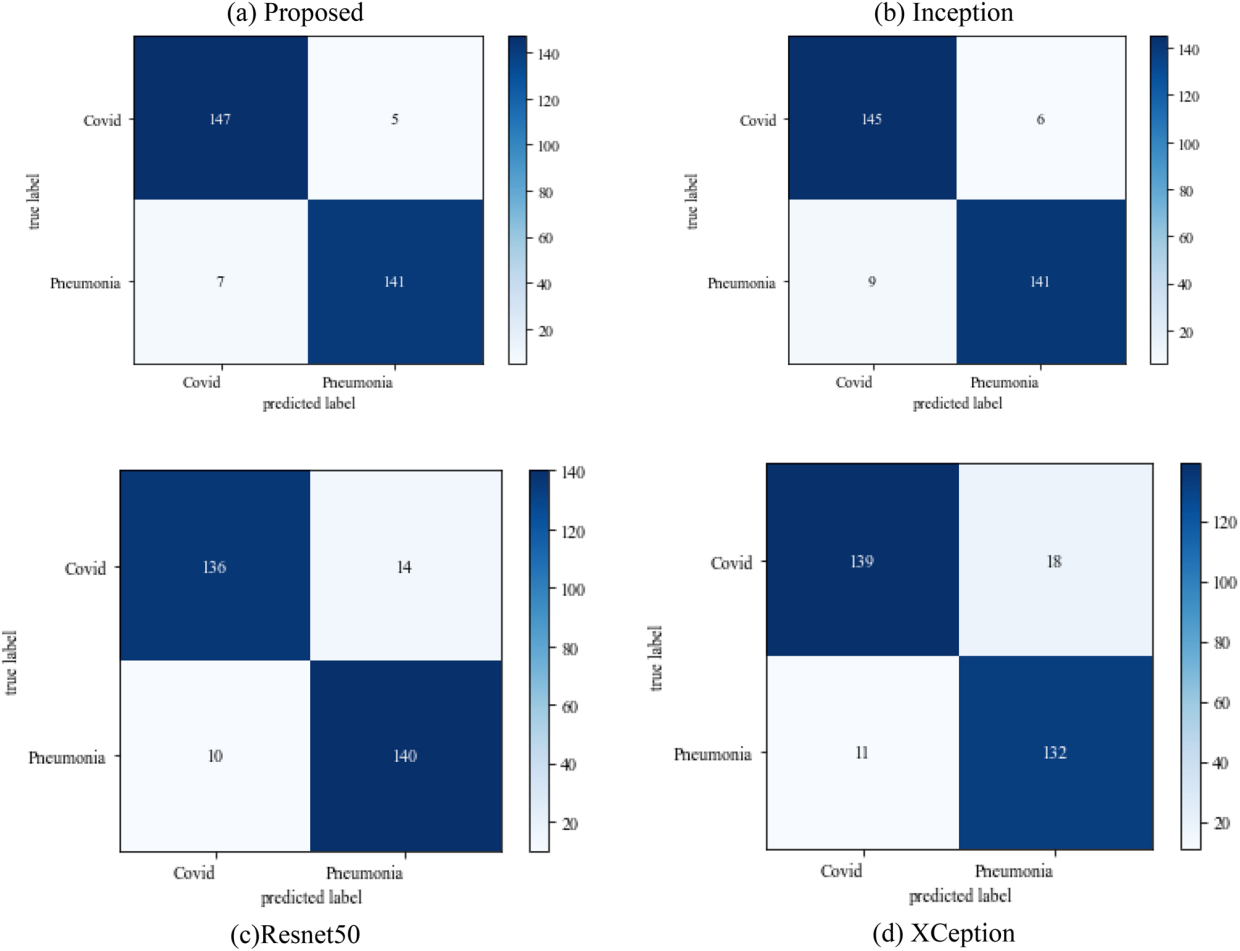
Confusion matrix on scenario 1.

The following results are based on scenario 1, i.e., dataset ratio of 70:30 in which 70% of images belong to the training set and 30% belong to the testing dataset. After hyperparameter tuning step, the suggested approach is compared to other models. Figure 8 represents the confusion matrix.

**Figure 9:**
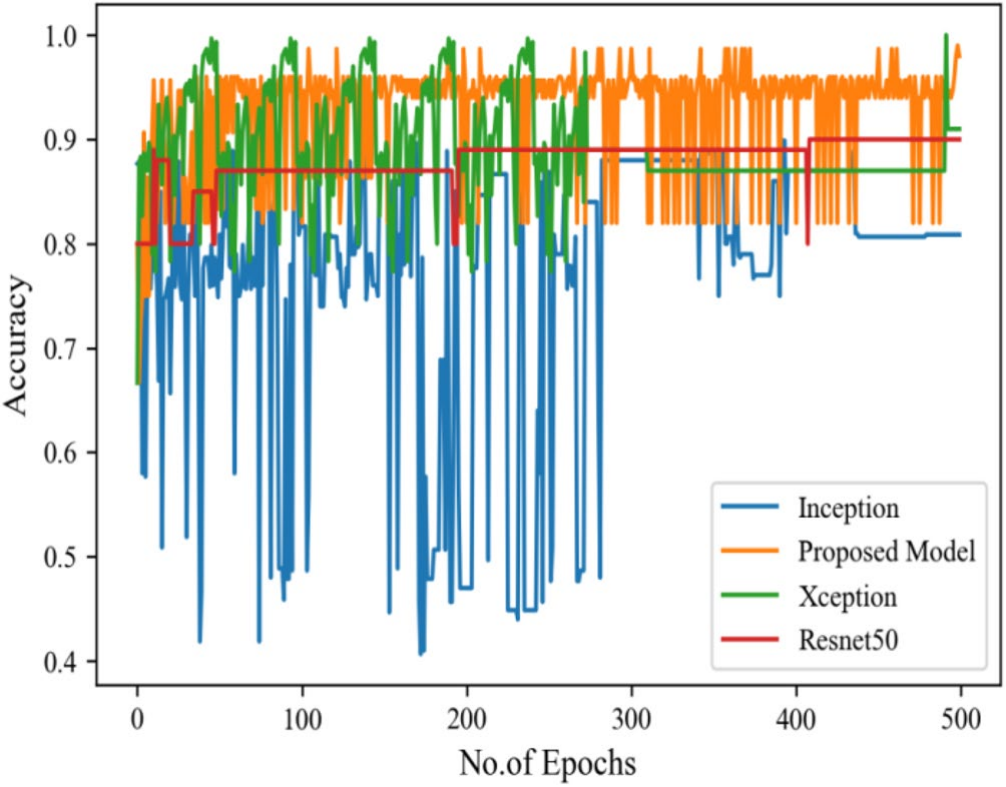
Testing accuracy with 500 epochs.

**Figure 10:**
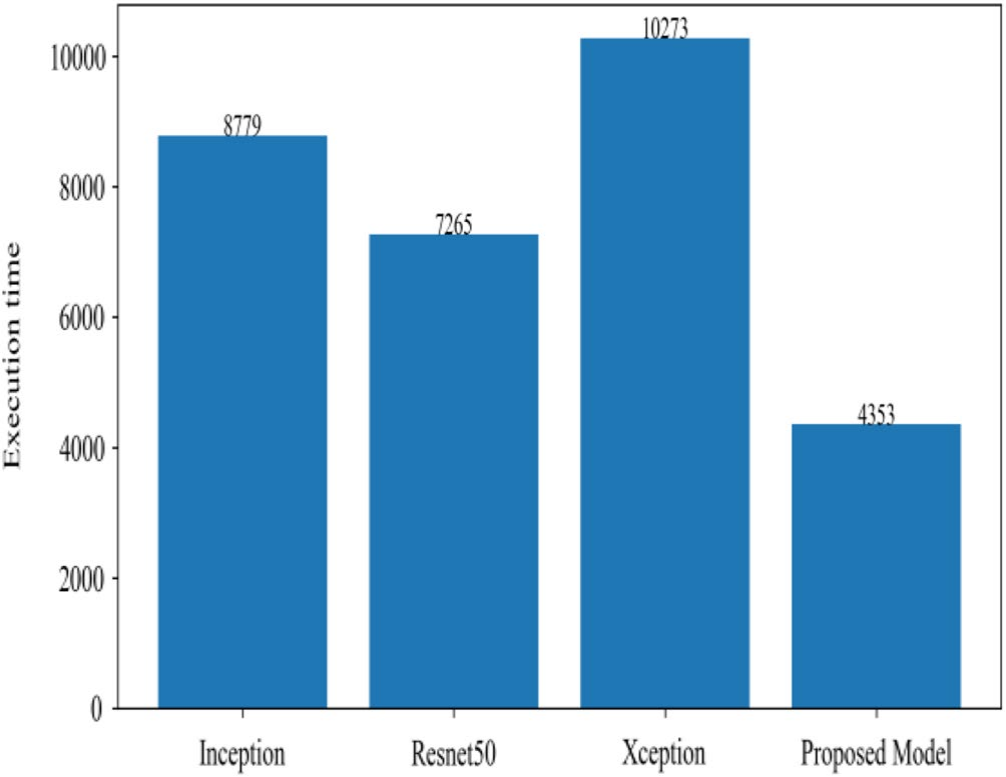
Execution time of each model.

Figure 8 represents the confusion-matrix of bifurcation of the dataset. Figure 9 represents the percentage of success for classifying COVID disease in 500 epochs. The proposed model gives 96% accuracy which is better than other models. The bar graph representation in Fig. 10 sum up the time of execution. The proposed approach takes less time as contrast to other models because it is lightweight and used fewer parameter which makes it faster than other models.

**Figure 11:**
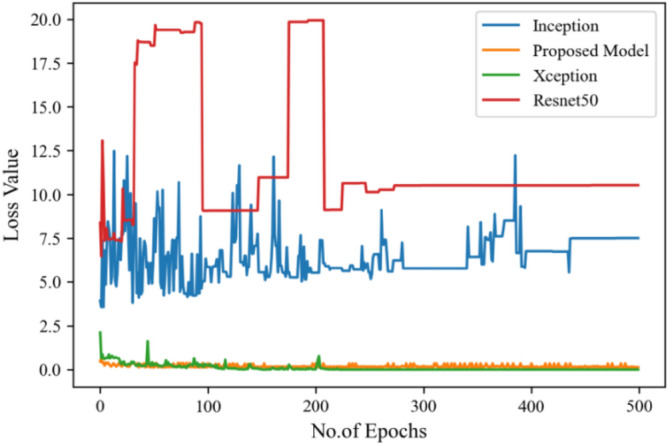
Testing loss with 500 epochs.

Moving ahead, Table 5 shows the performance metrics obtained in scenario 1, containing the value of F1-score, recall and precision. In this table, the proposed model shows the highest value of F1-score, recall and precision for class COVID and viral-pneumonia. Figure 10 represents the testing loss of each model with 500 epochs. The loss value shows how much error rate is there in the model performance, the resulting graph shows that Resnet50 has high value of loss rate as compared to other models. Figure 11 illustrates the testing loss with 500 epochs for all models.

**Table 5:** Model results with scenario 1.

Model	Labels	Precision	Recall	F1-score
InceptionV3	COVID-19	0.96	0.89	0.92
	Viral_pneumonia	0.90	0.96	0.93
Resnet50	COVID-19	0.90	0.89	0.89
	Viral_pneumonia	0.85	0.88	0.86
Xception	COVID-19	0.90	0.86	0.88
	Viral_pneumonia	0.92	0.91	0.91
Proposed model	COVID-19	0.97	0.95	0.96
	Viral_pneumonia	0.95	0.97	0.96

#### Scenario 2

**Figure 12:**
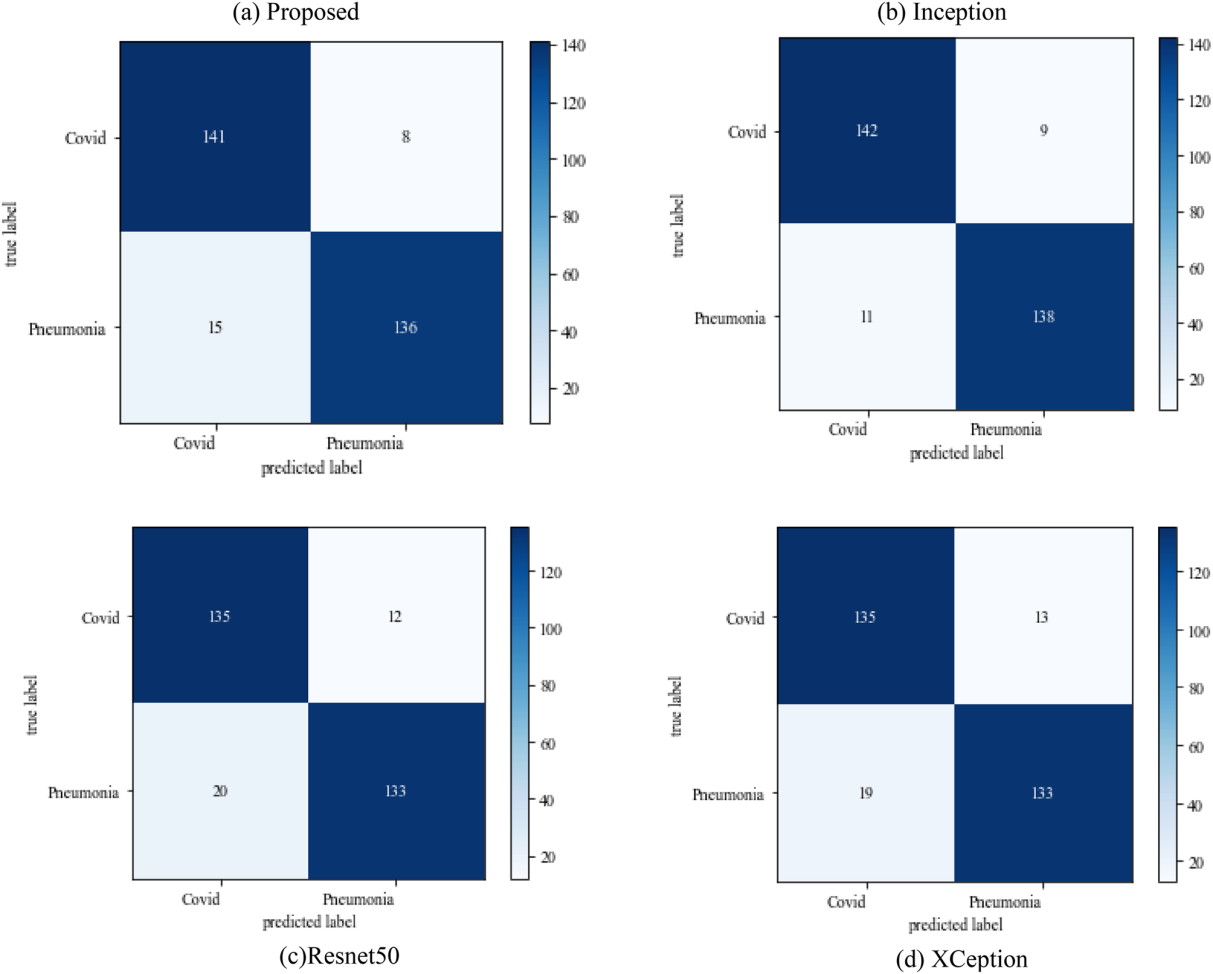
Confusion_matrix on scenario-2.

The following results are based on scenario 2 with a dataset ratio of 60:40 in which 60% of images belong to the training dataset and 40% belong to testing dataset. Figure 12 shows a confusion matrix that illustrates that the proposed model and Inception model have high true positive value i.e. 141 and 142 as compared to other models. In the confusion matrix true positive value of proposed model is 141 which means the COVID images are correctly classified as COVID and 136 as true negative value which shows the viral pneumonia images correctly classified.

Table 6 displays the f1-score, recall and precision value of models. The model outperforms the Inception model in precision and surpasses the Xception model in recall. The projected model gives the maximum value of precision and recall which makes it better than other models.

**Table 6:** Model results on scenario 2.

Model	Labels	Precision	Recall	F1-score
InceptionV3	COVID-19	0.93	0.92	0.92
	Viral_pneumonia	0.92	0.94	0.93
Resnet50	COVID-19	0.84	0.87	0.85
	Viral_pneumonia	0.89	0.86	0.87
Xception	COVID-19	0.94	0.91	0.92
	Viral_pneumonia	0.92	0.94	0.93
Proposed model	COVID-19	0.96	0.95	0.95
	Viral_pneumonia	0.95	0.96	0.95

**Figure 13:**
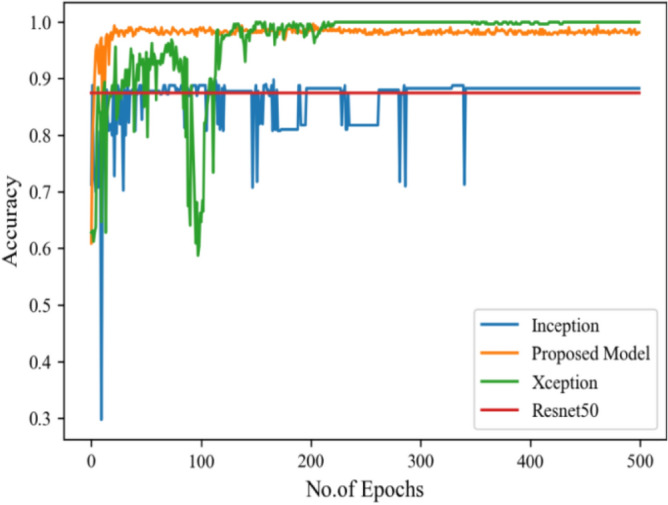
Testing accuracy with 500 epochs.

**Figure 14:**
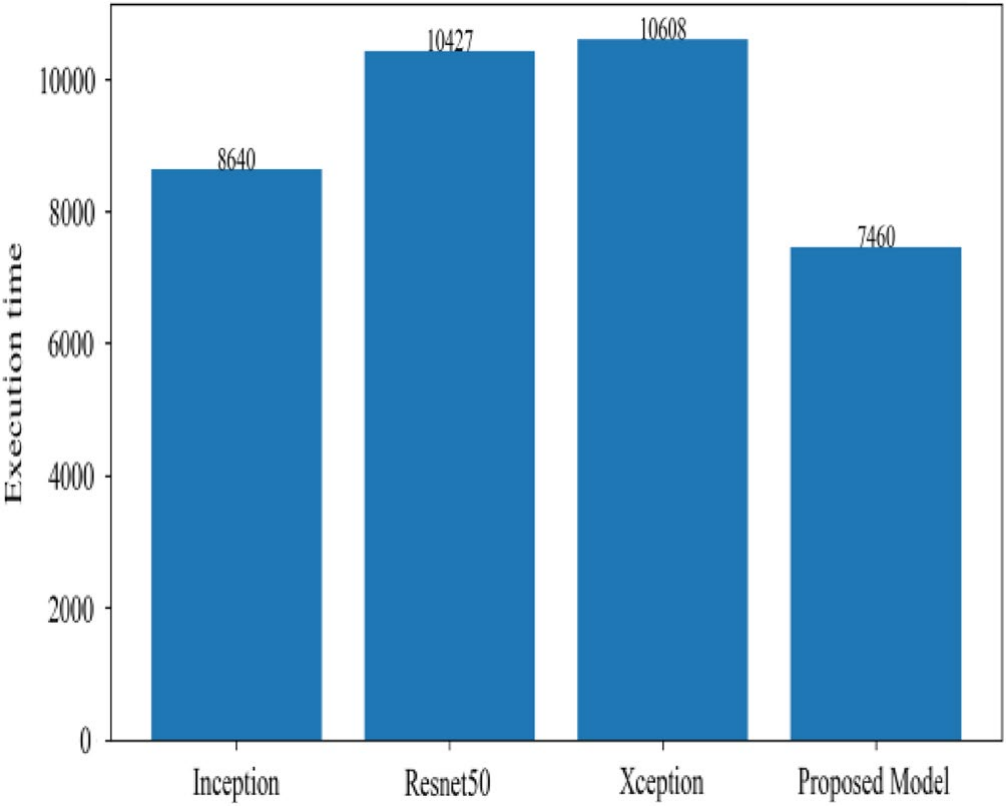
Execution time by each model.

Figure 13 represents the result of truly and correctly classified images of viral-pneumonia and COVID. The testing accuracy with Resnet50, Inception and XCeption is less as compared to the proposed model. Figure 14 displays the bar graph of the time executed by each model during the execution of the 500 epochs.

**Figure 15:**
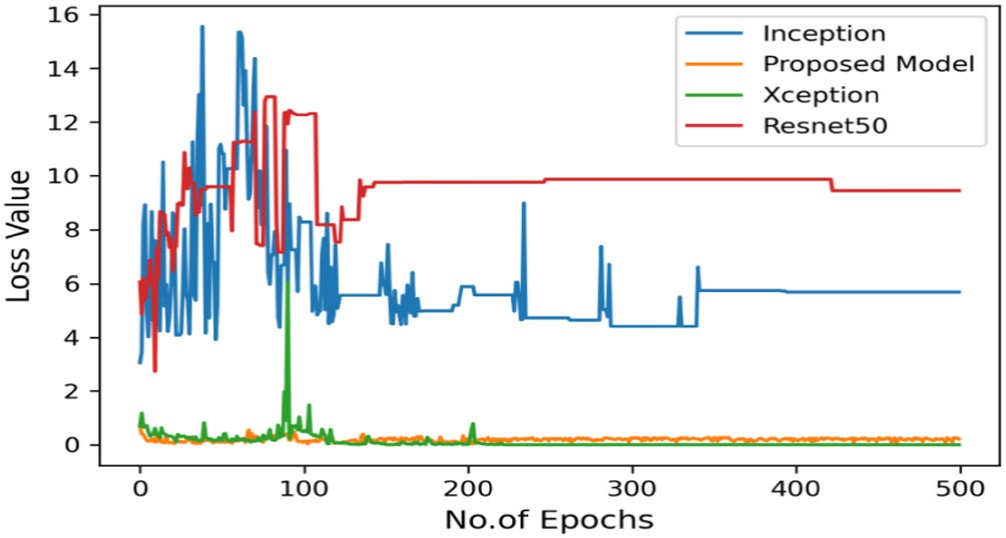
Testing loss with 500 epochs.

The proposed model was executed in 7460 s in total which is less than other transfer learning CNN models. The proposed model was executed in less time because the model architecture have lesser parameters as compared to other models. Figure 15 shows the result of validation loss rate with Resnet50, Inception and Xception. Loss defines how many the wrong predictions were made by the model. The proposed model gives less value of loss rate as compared to other models.

#### Scenario 3

**Figure 16:**
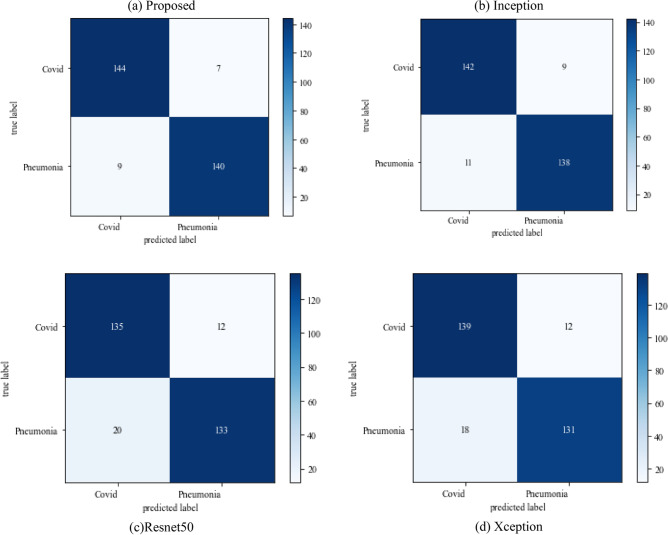
Confusion matrix on scenario 3.

The following results are based on scenario 3 on the dataset ratio 80:20 in which 80% of images belong to the training dataset and 20% belong to testing dataset. The confusion matrix which shows the result of model performance in predicting true images of diseases has been illustrated in Fig. 16

**Figure 17:**
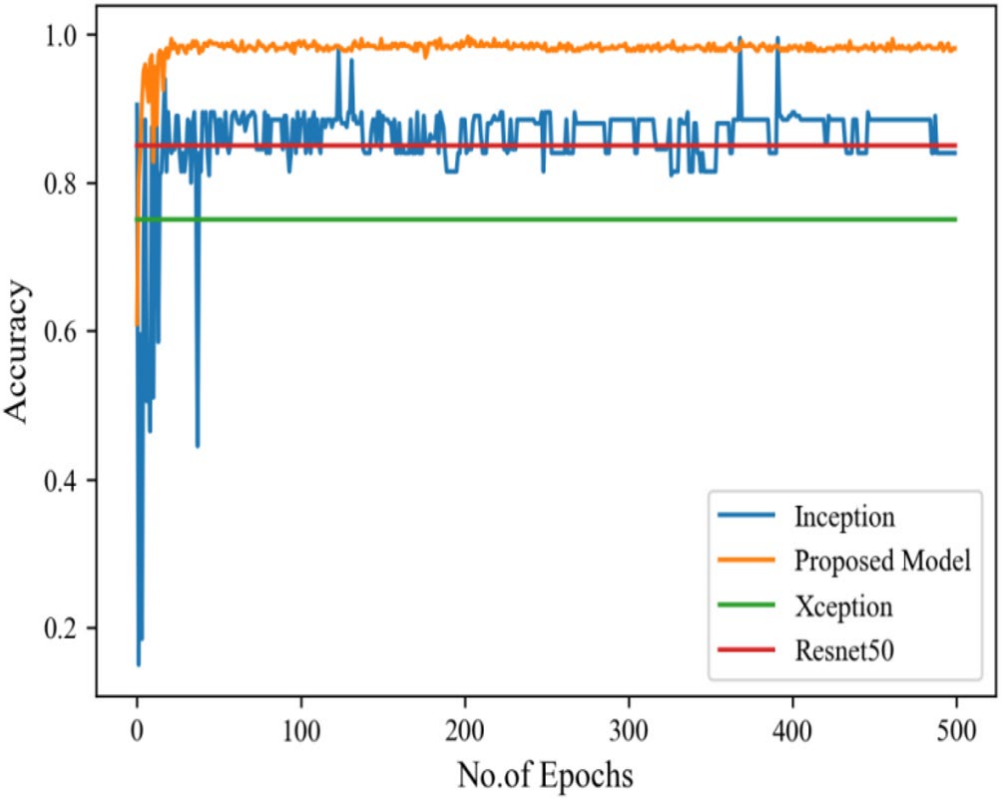
Testing accuracy with 500 epochs.

Figure 16 depicts the findings of the analysis confusion matrix, as well as the values related to performance metrics of the transfer learning model as well as suggested model. Table 7 displays the significance of the findings in terms of accuracy, recall, and f1-score of Inceptionv3, Resnet50, Xception, and the proposed model while using Scenario 3. Figure 17 represent the increasing success rate of classified data of proposed model as compared to three models with every epoch. The testing accuracy of Resnet50 is 0.89, Inception is 0.93, Xception is 0.74 and the proposed model is 0.96.

**Table 7:** Model results on scenario 3.

Model	Labels	Precision	Recall	F1-score
InceptionV3	COVID-19	0.92	0.91	0.91
	Viral_pneumonia	0.90	0.96	0.93
Resnet50	COVID-19	0.90	0.86	0.88
	Viral_pneumonia	0.85	0.88	0.86
Xception	COVID-19	0.91	0.89	0.90
	Viral_pneumonia	0.92	0.94	0.93
Proposed model	COVID-19	0.96	0.97	0.96
	Viral_pneumonia	0.95	0.96	0.95

**Figure 18:**
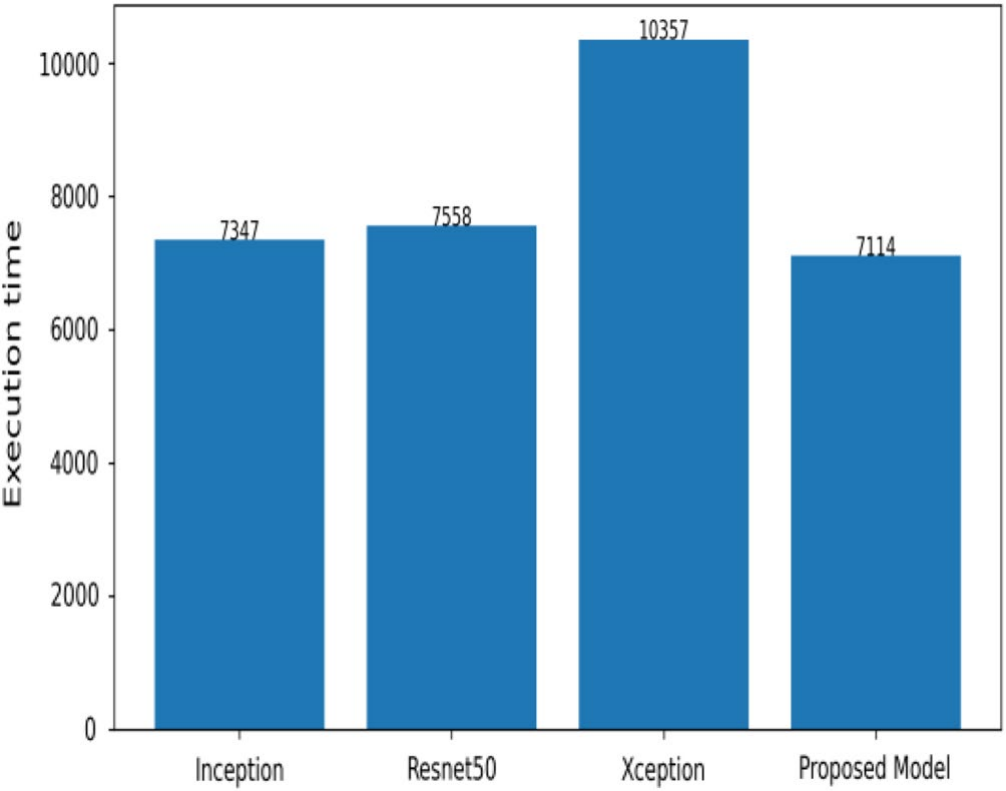
Execution time by each model.

**Figure 19:**
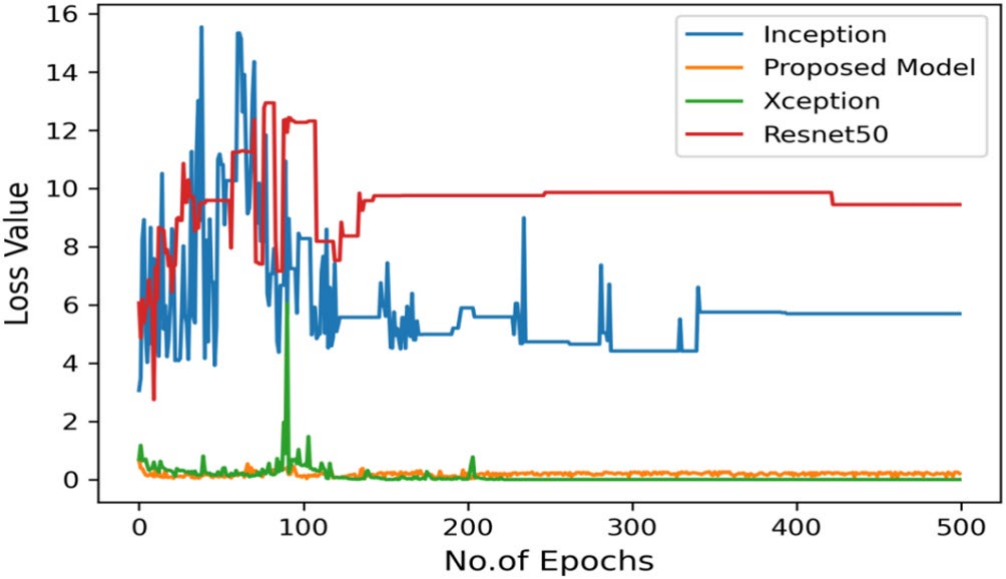
Validation loss with 500 epochs.

Figure 18 shows the bar graph of the time which define the completion of task by each model in 500 epochs. This bar graph shows the results of each model with 80:20 ratio dataset. The proposed model executed in 7114 s in total. Figure 19 shows the result of wrongly classified images rate by each model and the graph represent the different peak of loss rate with each epoch. The Resnet50 have highest value of loss as compared to other models.

### Experimental results with image enhancement

The following results are taken with Gaussian Blur images for $500$ epochs, and maximum accuracy of $98\%$ was observed. With every epoch, the accuracy rate of our proposed model with image enhancement gets improved. The functioning of CNN model with image enhancement images got improved in every epoch, the model uses smoothed and less noise image which increase its accuracy from 96 to 98%.

#### Scenario 1

The following results are based on scenario 1, i.e., dataset ratio of 70:30 in which 70% of images belong to the training set and 30% belong to the testing part of image enhancement dataset.

**Figure 20:**
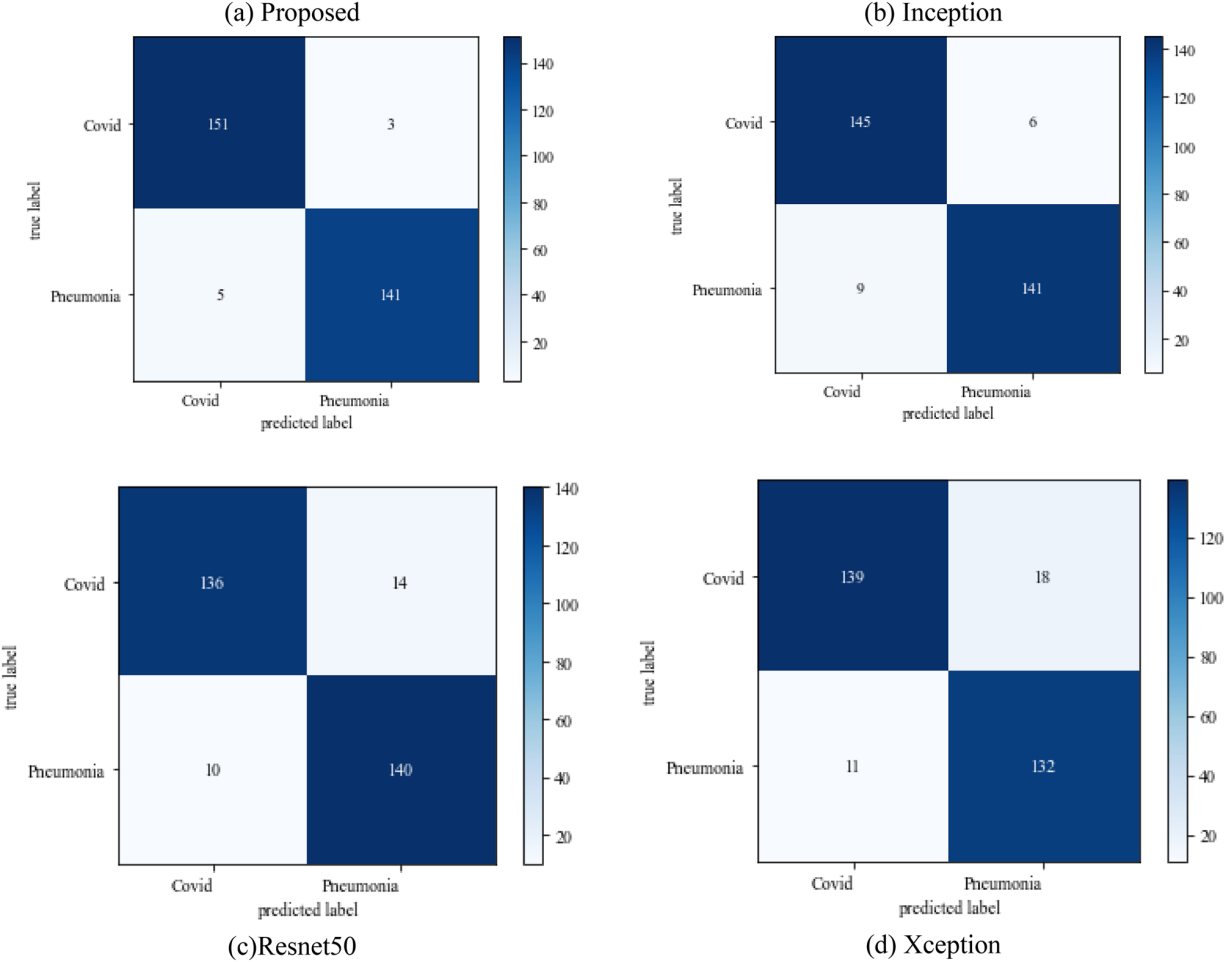
Confusion matrix on scenario 1.

**Figure 21:**
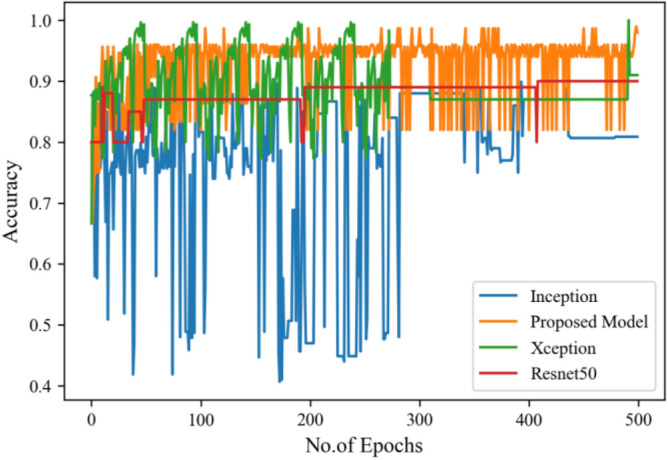
Testing accuracy with 500 epochs.

**Figure 22:**
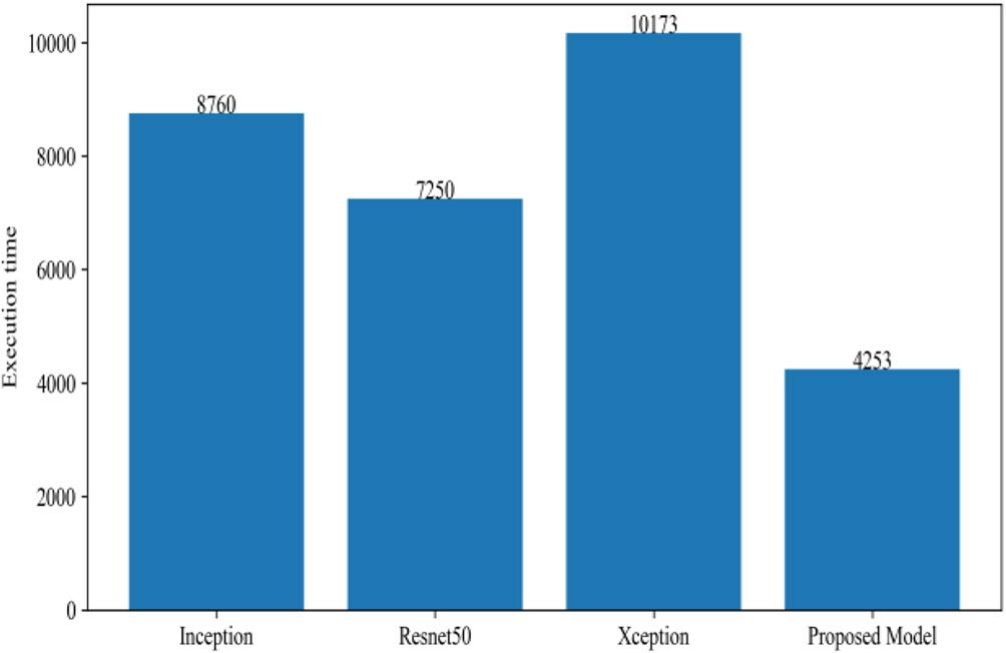
Execution time by each model.

Figure 20 shows the confusion matrix of image enhancement dataset in scenario 1. The confusion matrix shows the truly identified images by model and help in deep analysis of model performance. Figure 21 represents the true positive and true negative correctly identified classes of dataset in 500 epochs. The proposed model gives 98% accuracy which is better than other model's accuracy. The bar graph shows in Fig. 22 show the execution time of the model. The proposed model architecture contains less parameter which decrease the execution time.

**Figure 23:**
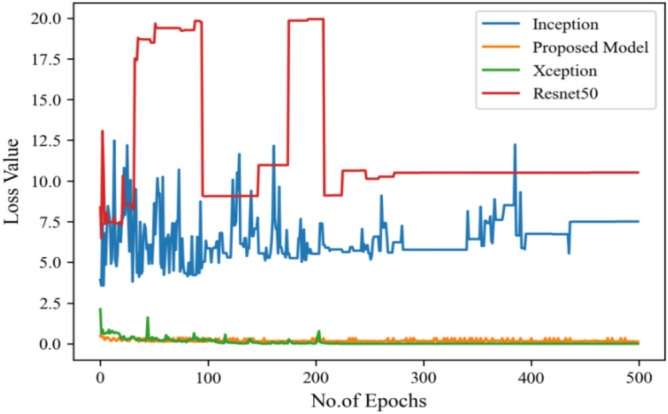
Testing loss with 500 epochs.

Moving ahead, Table 8 shows performance metrics obtained in scenario 1. Accuracy can be misleading if used with imbalanced dataset and therefore the other metrics such as f1-score, recall and precision useful are for evaluation. Table 6 is the summarization of the results of each model. Figure 23 represents the loss rate of each model with 500 epochs. The Loss value shows how much error rate is there in the model’s performance, the resulting graph shows that Resnet50 has high value of loss rate as compared to other models.

**Table 8:** Model results with scenario 1.

Model	Labels	Precision	Recall	F1-score
InceptionV3	COVID-19	0.96	0.89	0.92
	Viral_pneumonia	0.90	0.96	0.93
Resnet50	COVID-19	0.93	0.92	0.92
	Viral_pneumonia	0.92	0.94	0.93
Xception	COVID-19	0.90	0.89	0.89
	Viral_pneumonia	0.85	0.88	0.86
Proposed model	COVID-19	0.97	0.97	0.97
	Viral_pneumonia	0.97	0.98	0.98

#### Scenario 2

**Figure 24:**
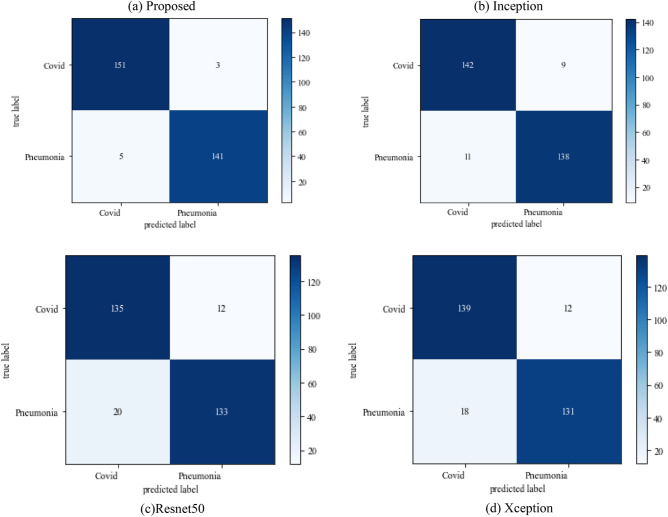
Confusion matrix on scenario 2.

The following results are based on scenario 2 with a dataset ratio of 60:40 in which 60% of images belong to the training dataset and 40% belong to testing dataset. The classifier matrix has been portrayed in Fig. 24, which shows the true positive value of proposed model is 151 which is high than others. Table 9 give the performance of models which shows the proposed approach model gives the better value of precision and recall which makes it better than other models.

**Table 9:** Model results in scenario 2.

Model	Labels	Precision	Recall	F1-score
InceptionV3	COVID-19	0.91	0.89	0.90
	Viral_pneumonia	0.92	0.94	0.93
Resnet50	COVID-19	0.90	0.89	0.89
	Viral_pneumonia	0.85	0.88	0.86
Xception	COVID-19	0.90	0.86	0.88
	Viral_pneumonia	0.92	0.91	0.91
Proposed model	COVID-19	0.96	0.97	0.96
	Viral_pneumonia	0.97	0.98	0.97

**Figure 25:**
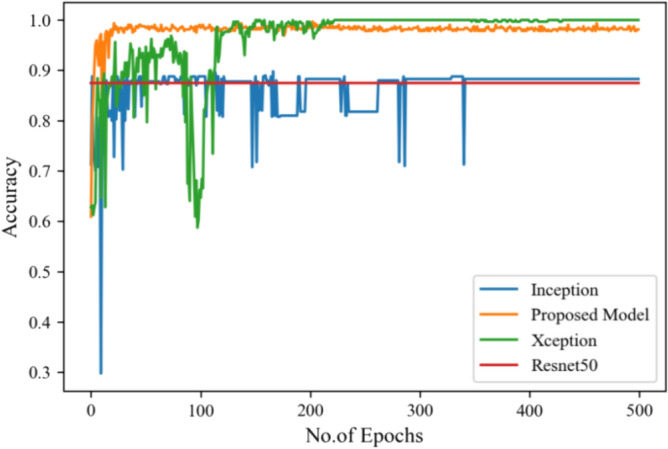
Testing accuracy with 500 epochs.

**Figure 26:**
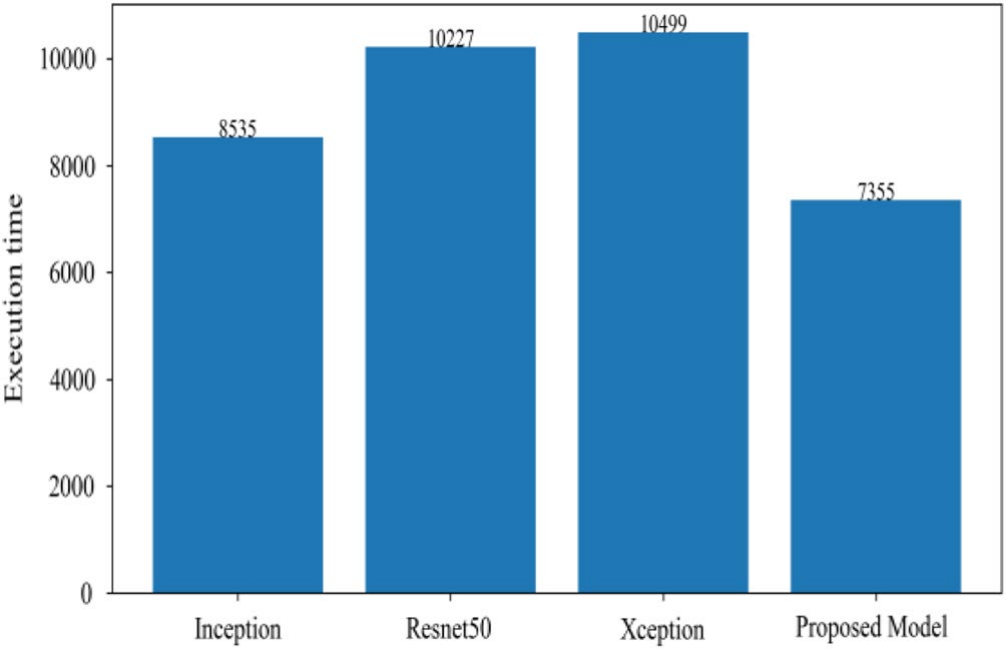
Execution time by each model.

**Figure 27:**
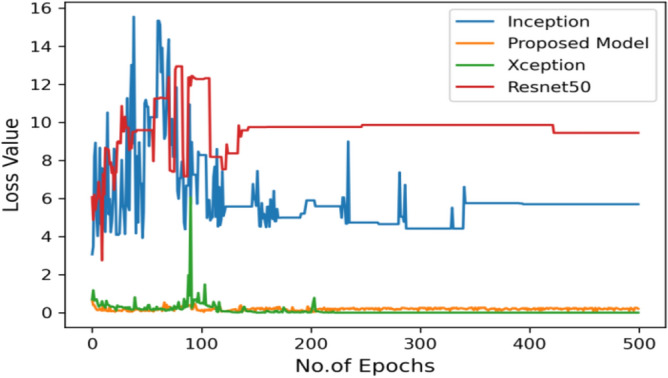
Testing loss with 500 epochs.

Figure 25 illustrated the percentage of success in classifying COVID and viral-pneumonia class correctly. The testing accuracy with Resnet50, Inception and XCeption is less as compared to the this paper model. It gives high classification result of 98% as compared to other models with image enhancement images. Figure 26 shows the bar graph of the time executed by each model during the execution of the 500 epochs. The proposed model was executed in 7335 s in total which is less than other CNN models. Figure 27 shows the result of validation loss and also the error rate. When pitted against other models, the proposed model exhibits a reduced loss rate.

#### Scenario 3

**Figure 28:**
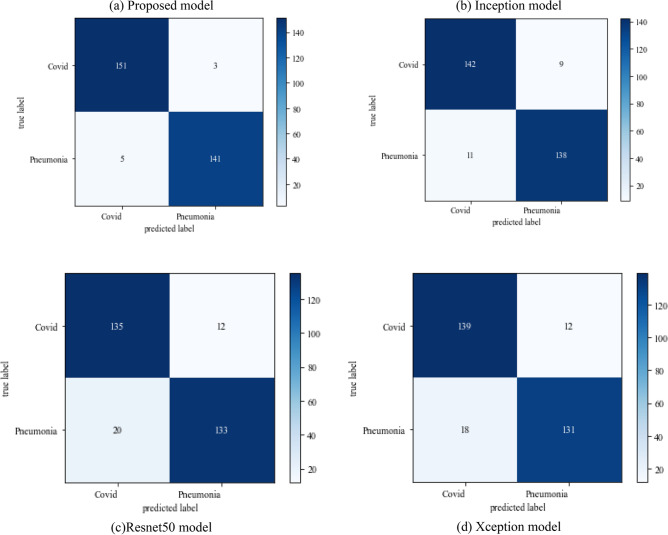
Confusion matrix on scenario 3.

The following results are based on scenario 3 on the dataset ratio 80:20 in which 80% of images are belonging to the training dataset and 20% belong to testing dataset. Figure 28 shows the confusion matrix which has obtained values of performance metrics of transfer learning model and proposed model.

**Figure 29:**
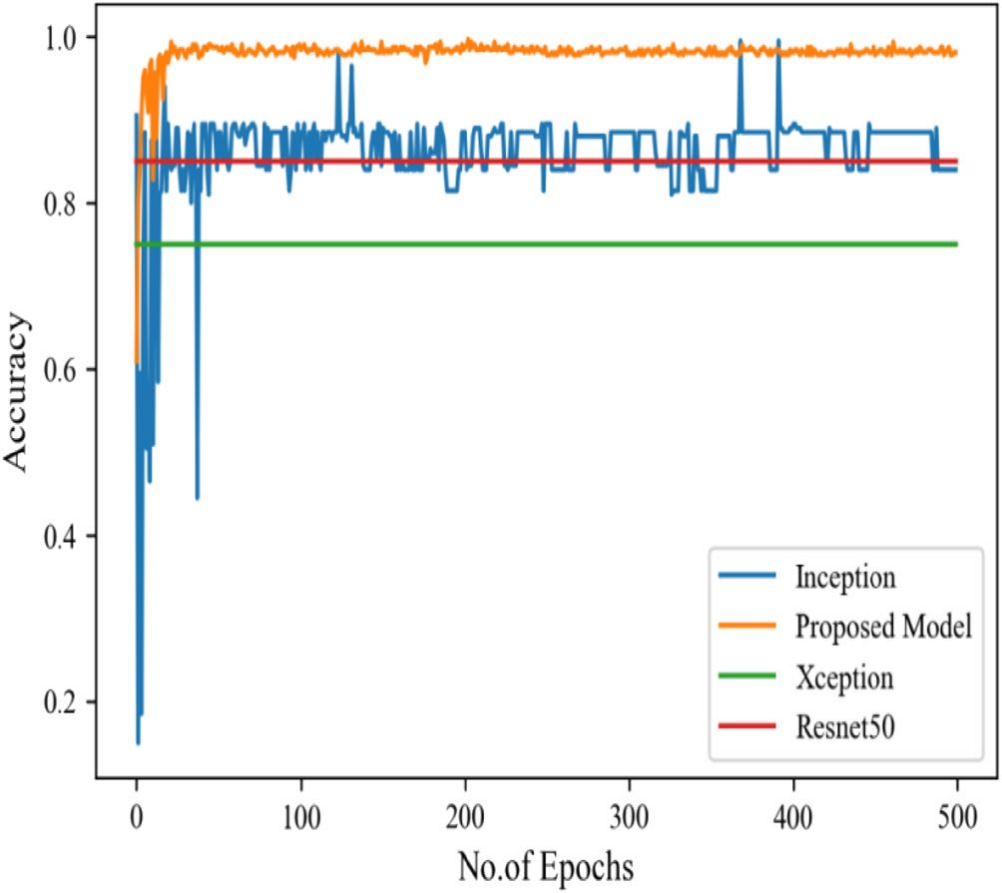
Testing accuracy with 500 epochs.

**Figure 30:**
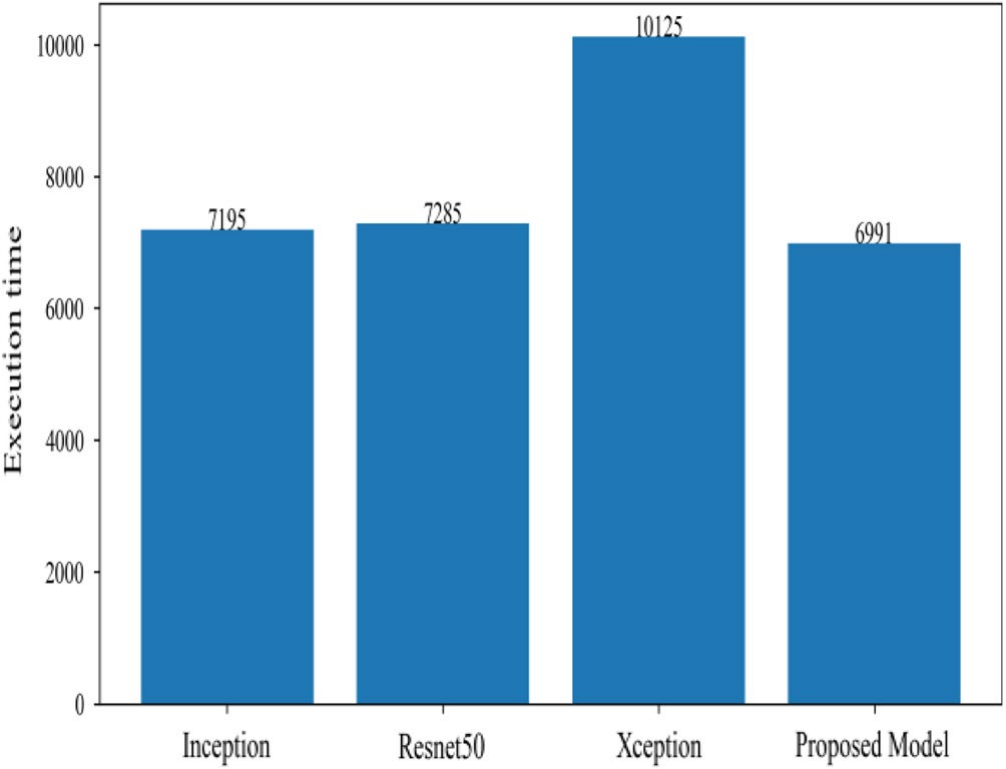
Execution time by each model.

Table 10 shows the precision, recall and f1-score values of Inceptionv3, Xception Resnet50 and the proposed model on the dataset in scenario 3 with 80% of the photos are for training, while 20% are for testing. Figure 29 represents that the testing accuracy of Resnet50 is 0.89, Inception is 0.93, Xception is 0.74 and the proposed approach is 0.98. When compared to other models, the Xception produces lower value results. Figure 30 shows the execution time of each model in 500 epochs.

**Table 10:** Model results on scenario 3.

Model	Labels	Precision	Recall	F1-score
InceptionV3	COVID-19	0.92	0.91	0.91
	Viral_pneumonia	0.90	0.96	0.93
Resnet50	COVID-19	0.90	0.86	0.88
	Viral_pneumonia	0.85	0.88	0.86
Xception	COVID-19	0.91	0.89	0.90
	Viral_pneumonia	0.92	0.94	0.93
Proposed model	COVID-19	0.98	0.97	0.97
	Viral_pneumonia	0.98	0.98	0.98

**Figure 31:**
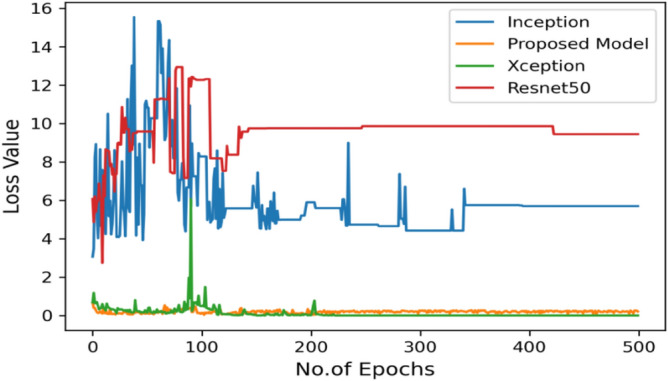
Validation loss with 500 epochs.

Figure 31 exhibits the validation loss outcome along with the result analysis, providing the count of actual and predicted labels for all classes.

**Figure 32:**
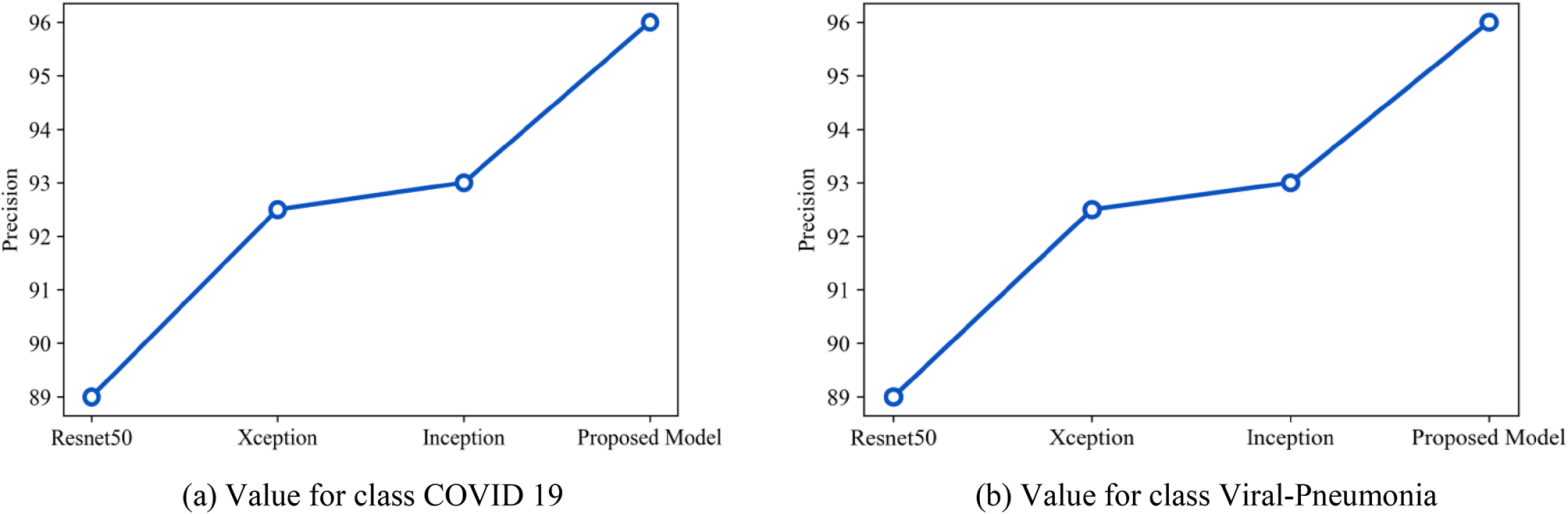
(**a**) Value for class COVID 19, (**b**) value for class viral-pneumonia.

Precision values show that out of all the non-COVID 19 infected cases, how many were identified correctly by the model. Figure 32 shows the overall precision value of Resnet50, Inception, Xception and the proposed approach. Figure 32a represents the values for class COVID 19 in Resnet50 is 88, Inception gives a value of 93.6, Xception gives a value of 91.6 and proposed model is 96.3. Figure 32b shows the values for class Viral pneumonia as Resnet50 is 86.5, Inception gives a value of 90.6, Xception gives a value of 92 and the proposed model is 97.

### Aggregation of three scenarios

This section presents the average of all the results obtained in the above mentioned three scenarios. The results are the average of the model which includes the three phases: image enhancement, data augmentation and hyperparameter tuning. The average value of recall, precision, f1-measure and accuracy based on the average of all results with image enhancement dataset.

**Figure 33:**
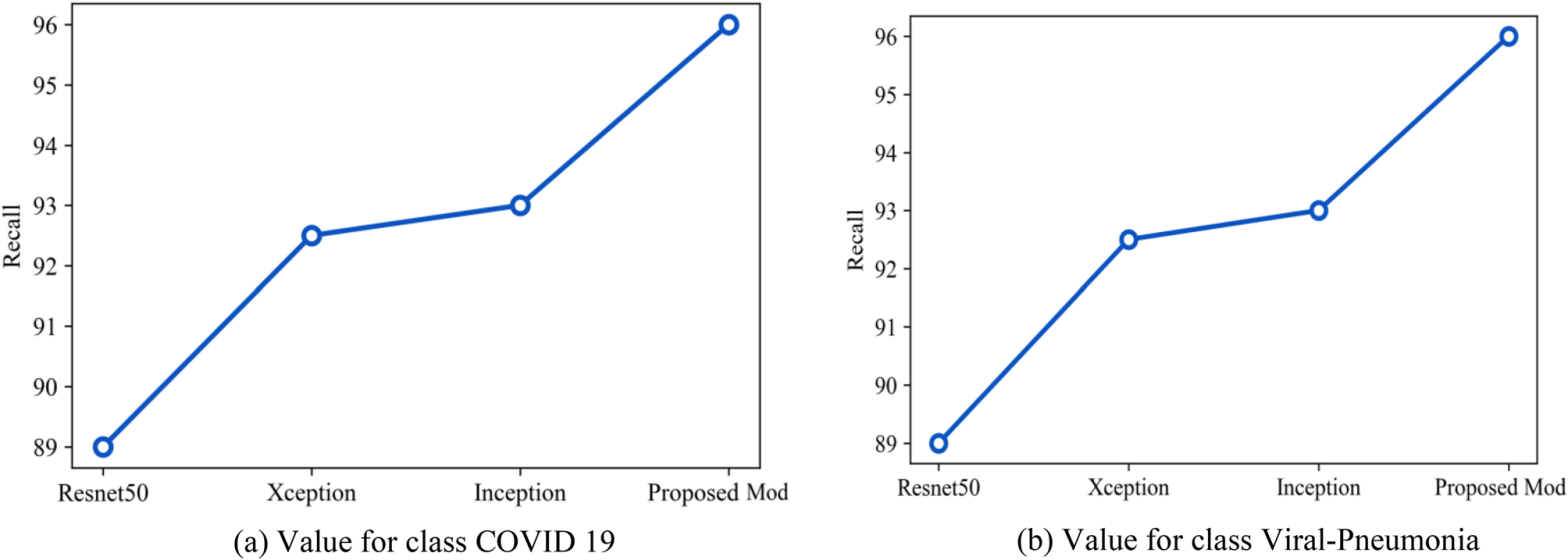
(**a**) Value for class COVID 19, (**b**) value for class viral-pneumonia.

Recall value shows out of all the COVID 19 patients, how many were identified correctly by the model. Figure 33 shows the overall recall value (in percentage) of Resnet50, Inception, Xception and the proposed approach. Figure 33a represents the values for class COVID 19 as Resnet50 is 95.6, Inception gives a value of 90, Xception gives a value of 88.6 and proposed model is 95.3. Figure 33b shows the values for class Viral pneumonia as Resnet50 is 87, Inception gives a value of 95, Xception gives a value of 93 and proposed model is 98.

**Figure 34:**
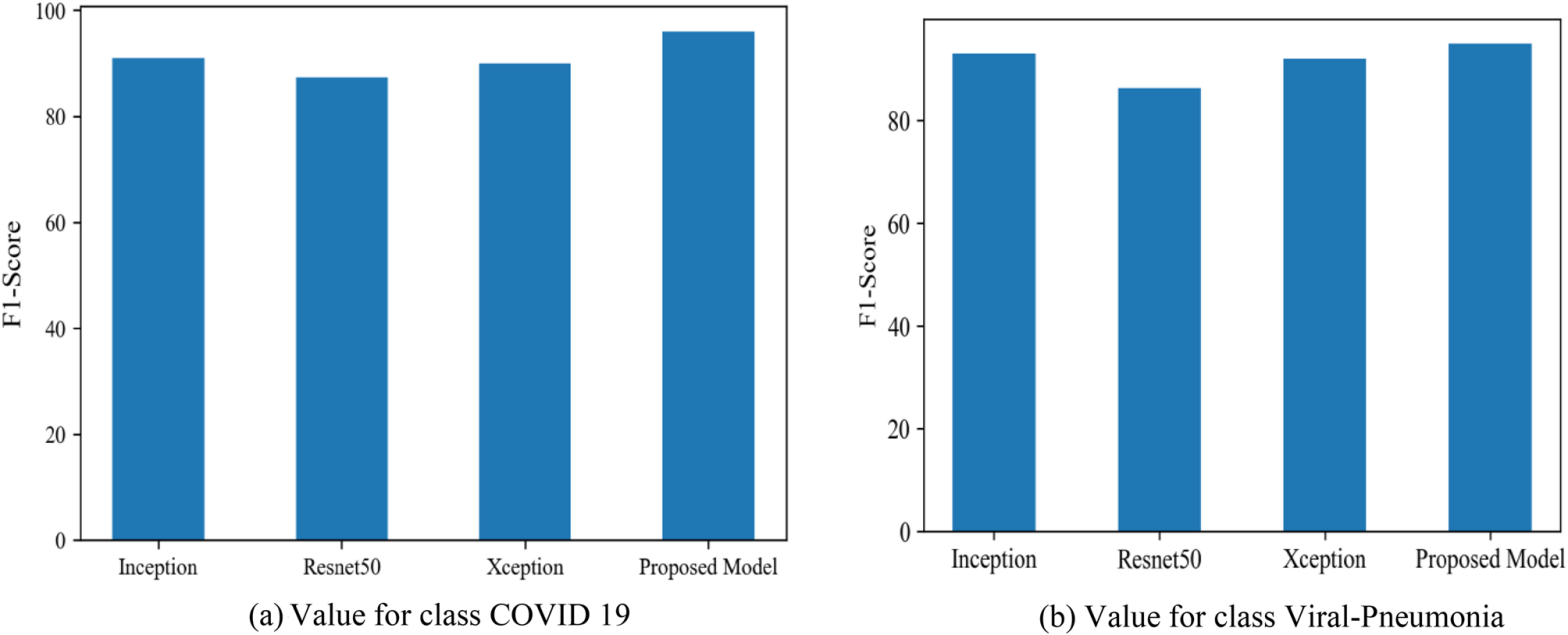
(**a**) Value for class COVID 19, (**b**) value for class viral-pneumonia.

Unfortunately, it is not feasible to maximize both accuracy and recall simultaneously. So, there is another metric available for convenience called F-measure. Figure 34a shows f1-score values for class COVID 19 as Resnet50 is 87.3%, Inception gives a value of 91%, XCeption gives a value of 90% and proposed model is 96%. Figure 34b shows the values for class Viral pneumonia as Resnet50 is 86.3%, Inception gives a value of 93%, XCeption gives a value of 92% and for the proposed model it is 97%.

**Figure 35:**
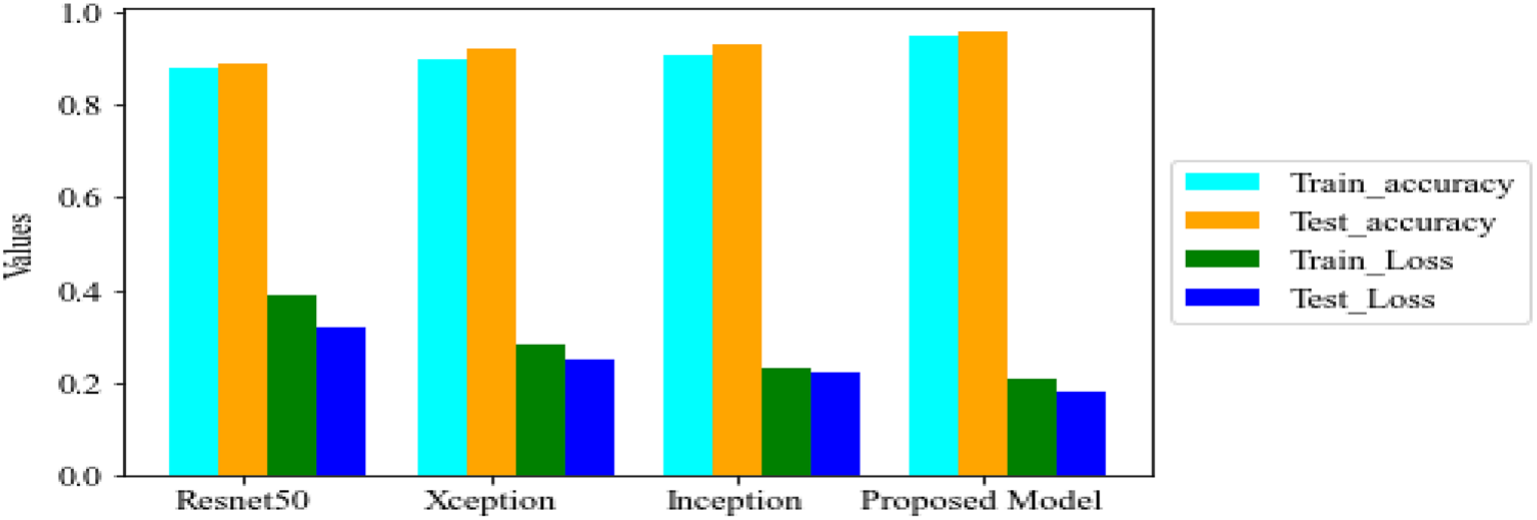
Average accuracy and loss.

**Figure 36:**
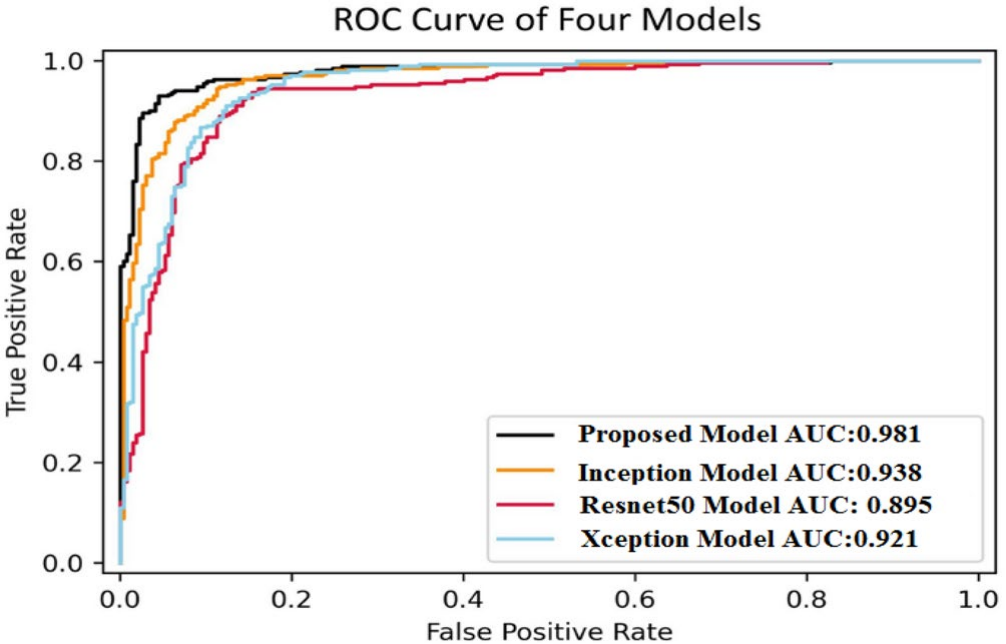
ROC curve of models.

Figure 35 shows the average accuracy of all models based on the different experiments. The average accuracy of Resnet50 is 0.89, Inception accuracy is 0.93, the accuracy of XCeption is 0.92 and proposed model accuracy is 0.98. The proposed model perform better in comparison to other models as shown in Figure 36 as ROC curve of models.

Table 11 represent the evaluation metrics such as sensitivity, specificity, positive and negative likelihood to enhance the comprehensibility of the model's performance. The proposed model give better results than other transfer learning models.

**Table 11:** Comparison table of models.

Model	Sensitivity	Specificity	Positive likelihood ratio	Negative likelihood ratio
Xception	0.915	0.918	11.56	0.0789
Resnet50	0.871	0.906	10.54	0.1256
Inception	0.928	0.935	15.16	0.0768
Proposed model	0.972	0.954	21.26	0.0285

It can be clearly gauged from the above result that the model proposed here is better than various other models in terms of various parameters. The outperformance of draft model can be credited to the various modifications to the CNN architecture, which can be further summarized by the following points.

**Figure 37:**
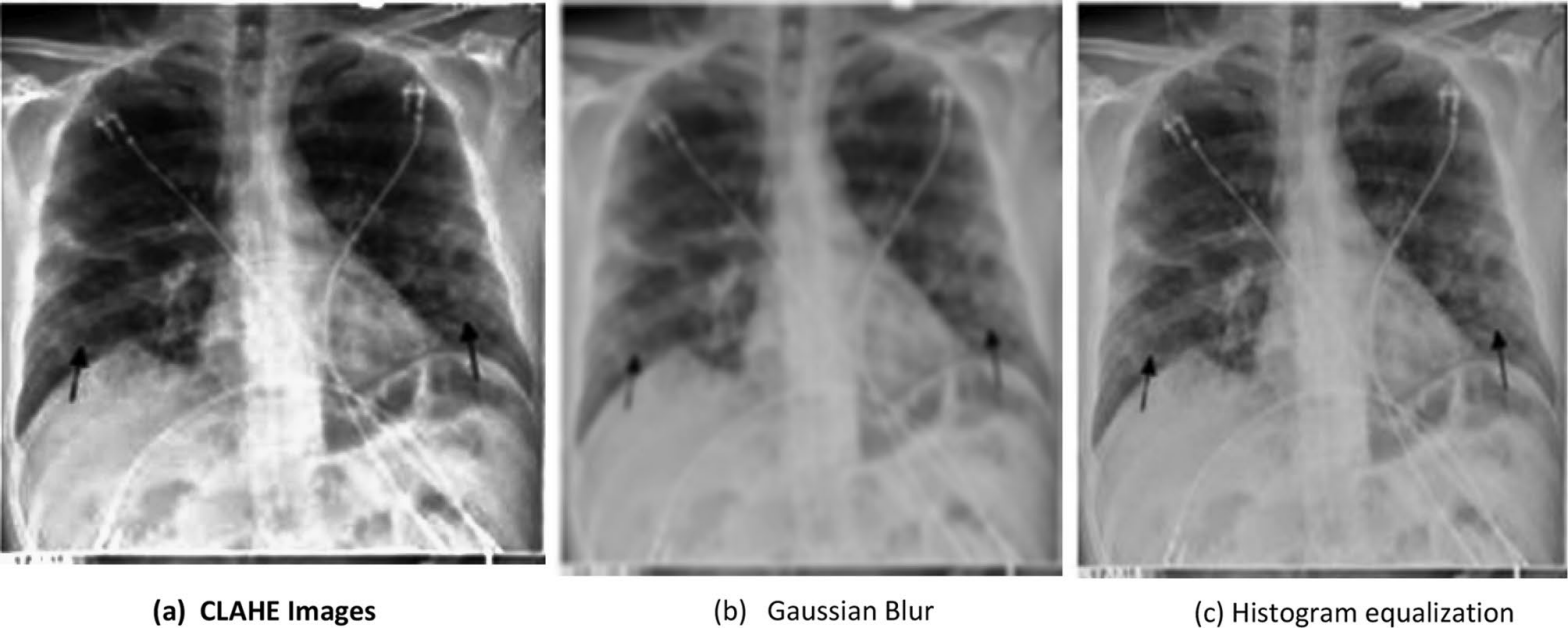
Image enhancement techniques based images.

1. The proposed model have considered three different image enhancement techniques that are Gaussian Blur, CLAHE, Histogram equalization. The Gaussian Blur removes the noise and intensity of images. Figure 37 show the image enhancement techniques images i.e. CLAHE, Gaussian Blur and Histogram equalization, out of which image enhancement using Gaussian blur technique leads to better classification accuracy as compared to other image enhancement techniques. The classification results obtained by using image enhancement using Gaussian blur is 98%, while image enhancement using CLAHE and histogram equalization give 94% and 93% classification accuracy, respectively.

Table 12, represent the comparative analysis of all three image enhancement techniques with parameters accuracy and loss rate.

**Table 12:** Comparative analysis of image enhancement techniques.

Enhancement technique	CLAHE images	Gaussian Blur	Histogram equalization
Accuracy	94%	98%	93%
Loss rate	0.65	0.23	0.74

**Figure 38:**
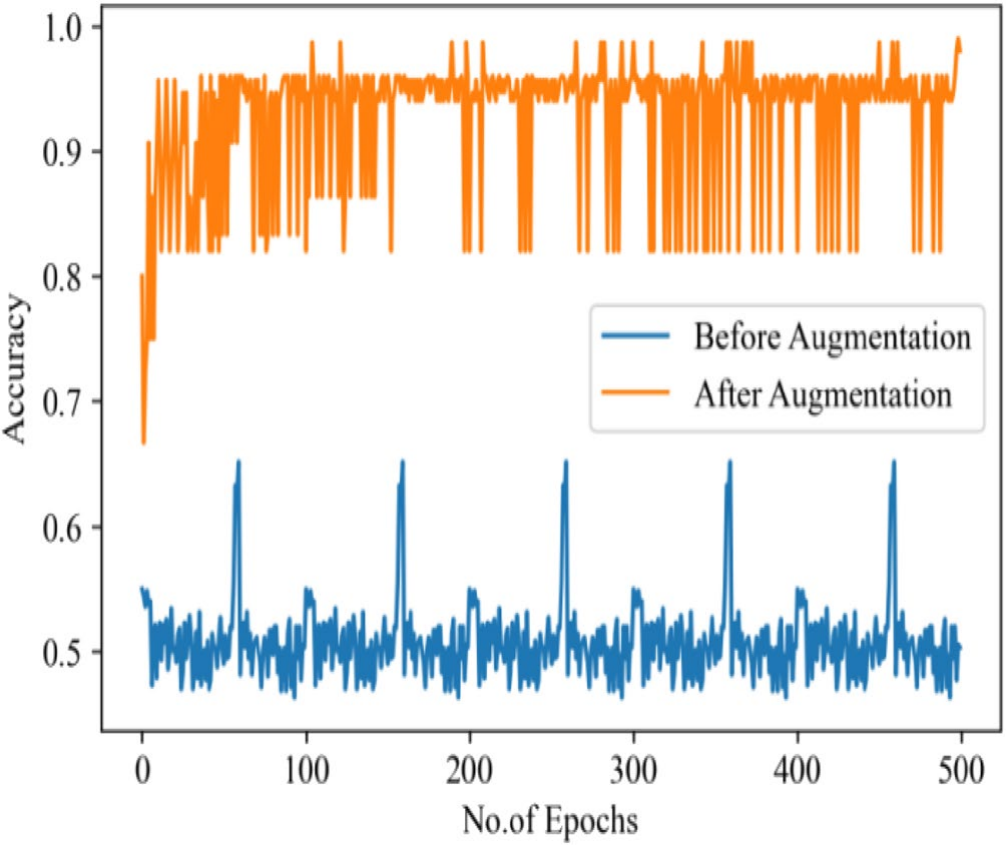
Accuracy value on 500 epoch.

**Figure 39:**
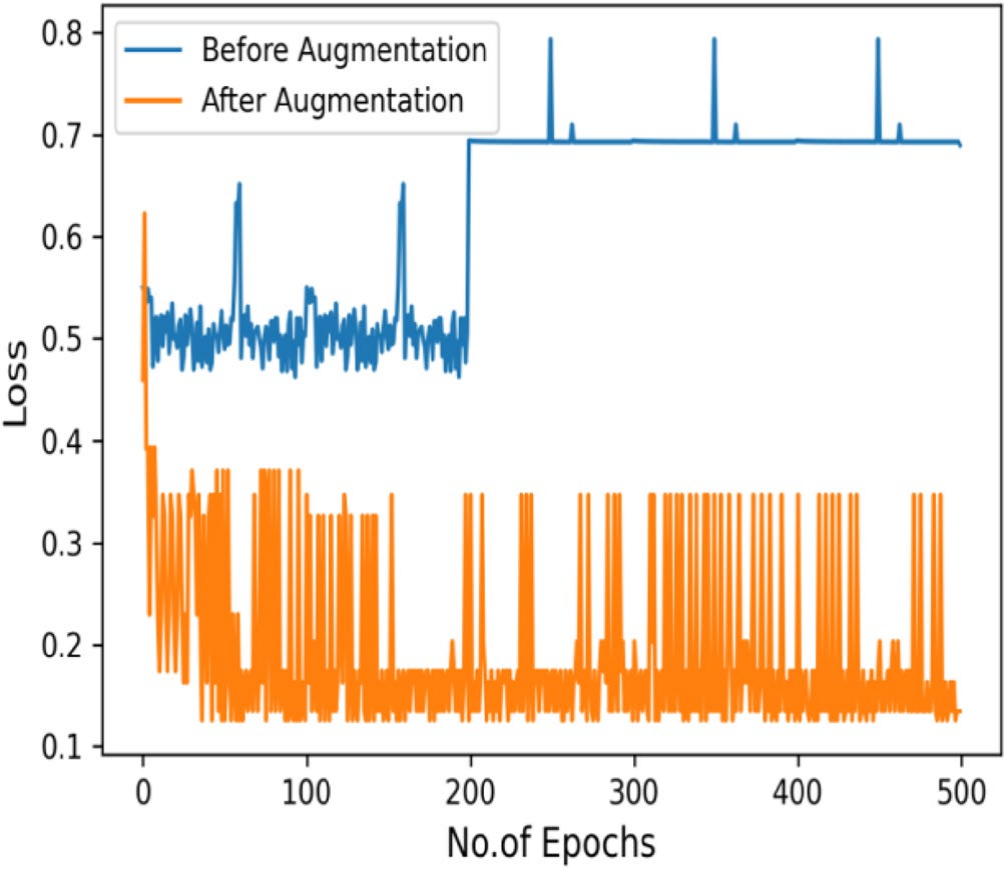
Loss value on 500 epoch.

2. Authors have also used the concept of data augmentation. It used to large the quantity data by adding slightly modify samples of data images. It can reduce the overfitting of model and also increase classification value by increasing the training data. The below figure clearly depicts the effect of image augmentation. The following two figures show the successful rate and loss rate of draft model before and after data augmentation. The accuracy of the model by using 500 epochs is shown in Fig. 38. And loss of value on 500 epochs is shown in Fig. 39.
3. And lastly, authors have tuned the hyperparameters and have made an effort to get the best parameters for increasing the precision and accuracy of classification. The hyperparameters^19^ are tuned to find better set of parameters fitted for the proposed model. The tuning of hyperparameters contain the number of neurons, epochs, optimizer, dropout, kernel size, no. of filters, learning rate and activation function. Table 13 shows the experiments conducted on the proposed model using two loss function i.e. Sparse-categorical cross_entropy (LF1) and Binary_cross_entropy (LF2) to adjust all the parameter in right direction. The loss function calculates the difference between the predicted values and the actual target values, providing a feedback signal to update the model's parameters through backpropagation and gradient descent. The choice of loss function depends on the desired behavior with respect to outliers in the data. Minimizing the loss implies that the model's predictions are becoming closer to the true values, leading to improved accuracy.

**Table 13:** Experiment for hyperparameters tuning.

Case No	Loss function	Optimizer	Dropout value	No. of layers (Conv)	Learning rate	Kernel size	Accuracy (%)
1	Sparse categorical cross entropy	RMSprop	0.4	3 layer	0.000001	3 × 3	96.6
2	Binary cross entropy	Adam	0.4	3 layer	0.000001	3 × 3	45
3	Sparse categorical cross entropy	Adam	0.4	3 layer	0.0001	3 × 3	96.3
4	Binary cross entropy	RMSprop	0.4	3 layer	0.000001	3 × 3	50
5	Sparse categorical cross entropy	SGD	0.4	3 layer	0.01	3 × 3	96
6	Binary cross entropy	SGD	0.4	3 layer	0.01	3 × 3	70
7	Binary cross entropy	Adam	0.7	3 layer	0.000001	3 × 3	42
8	Binary cross entropy	RMSprop	0.7	3 layer	0.0001	3 × 3	50
9	Sparse categorical cross entropy	Adam	0.7	3 layer	0.000001	3 × 3	42
10	Binary cross entropy	SGD	0.7	3 layer	0.01	3 × 3	50
11	Sparse categorical cross entropy	RMSprop	0.7	3 layer	0.0001	3 × 3	45
12	Sparse categorical cross entropy	SGD	0.7	3 layer	0.01	3 × 3	50

**Figure 40:**
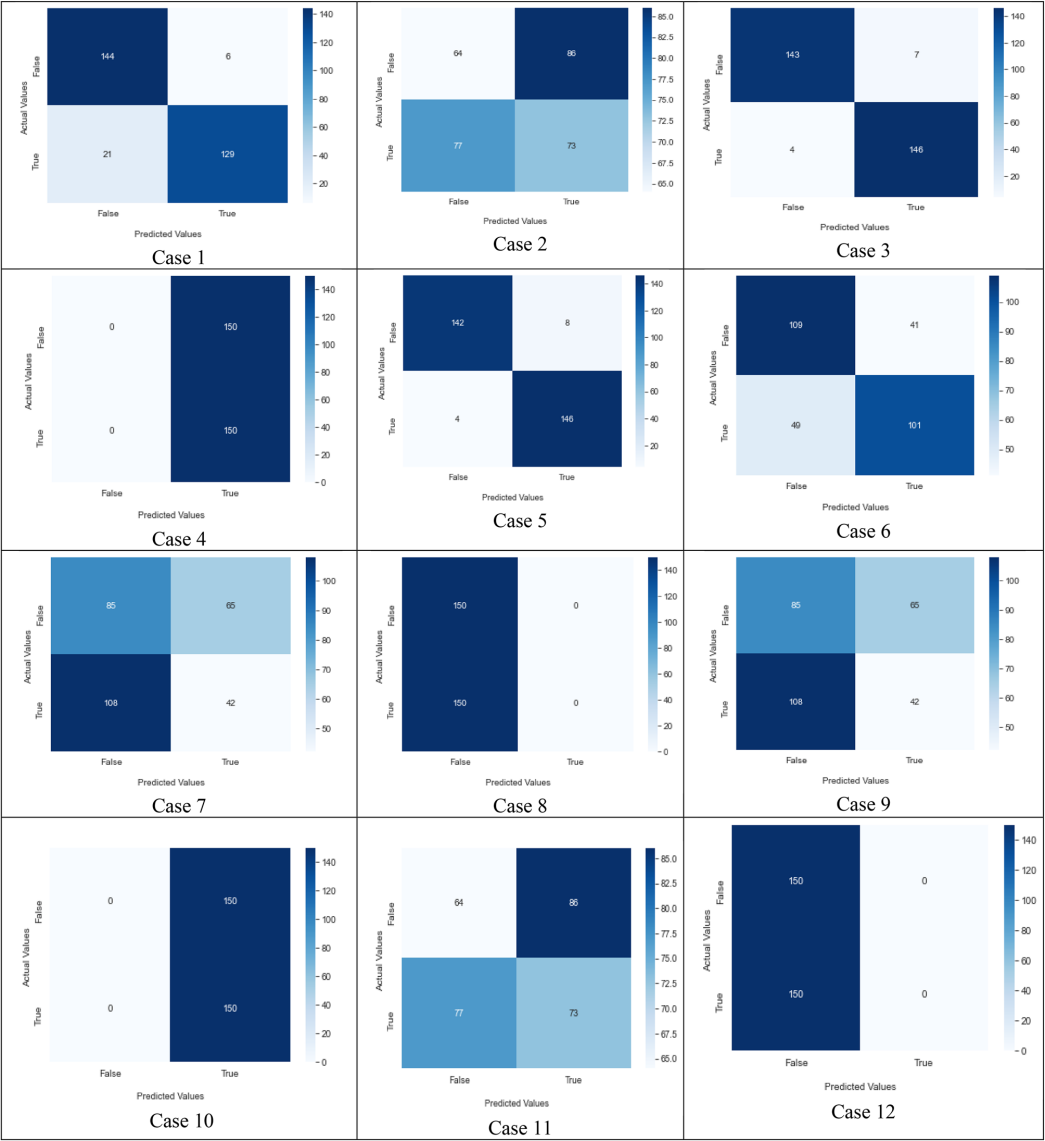
Confusion matrix of all hyperparameter tuning cases.

The confusion matrix of hyper parameter tuning cases is shown in Fig. 40. For the proposed model, The best hyperparameter case 3 selected for better accuracy rate. The results before hyper parameter tuning is 94% which is 2% less than the results after hyperparameter tuning.

### Comparative analysis of proposed model with state of the art approaches

Table 14 represents the performance comparison of our proposed model with existing approaches. It can be observed that the proposed model has outperformed all the other approaches.

**Table 14:** Comparative analysis of our proposed model with state of the art approaches.

Author	CNN Dark net^56^	VGG19^6^	Mobile Net v2^57^	Inception^58^	Xception Net^59^	Mobilenetv2 and VGG16^20^	Proposed model
Classes	COVID, No-Findings, Pneumonia	ILSVRC-2012 dataset 1000 classes	Multiple classes (car, person, bus etc.)	Multiple classes	Normal, pneumonia, and COVID-19	Normal, pneumonia viral, and COVID-19, pneumonia bacterial	COVID-19 and viral-pneumonia
Model	Dark Covid Net	Conv Net	Mobile Net Conv Mobile Net	Inception-v3	Xception and ResNet50V2	Mobilenetv2 and VGG16	Inception ,Xception and ResNet50
Accuracy	87.00%	93.48%	94.72%	92.85%	92.85%	96.4%	98.03%
Easy to train	✗	✗	✗	✗	✗	✗	✓
Avoids overfitting	✗	✗	✗	✗	✗	✗	✓
Fast training time	✗	✗	✗	✗	✗	✗	✓
Easy to generalize	✗	✗	✗	✗	✗	✗	✓
Less no. of layers	✗	✗	✗	✗	✗	✗	✓
Avoids vanishing gradient	✗	✗	✗	✗	✗	✗	✓
Works on slow devices	✗	✗	✗	✗	✗	✗	✓
Includes regularization	✗	✗	✗	✗	✗	✗	✓
Easy to distribute	✗	✗	✗	✗	✗	✗	✓

## Conclusion

In current times, deep learning models have come into existence and are playing a massive role in the development of various computer aided disease diagnosis systems. In this research, an attempt has been made to develop a concise CNN model by using lesser number of parameters (no. of layers, kernel size, optimizer, activation function) so as to reduce the execution time while obtaining a better classification accuracy for the diagnosis of COVID-19. The experiments have been performed on various X-ray images, using Gaussian Blur as an image enhancement technique and image augmentation in the image preprocessing stage. The performance of the proposed model is also compared with existing transfer learning models viz. Xception, Resnet50 and inception. The results clearly indicate the outperformance of the proposed model in terms of various parameters (accuracy, loss rate, precision, recall and f1-score). In near future, the proposed model can also be implemented for diagnosis of various other diseases and other different datasets of COVID-19. To reduce the dimensionality of data the various nature inspired meta-heuristic algorithms can be deployed to select the most dominant features while removing the redundant or less important features from the images so as to reduce the computation time and increasing the accuracy of classification.

## Data Availability

The X-ray of chest data is publicly available in https://www.kaggle.com/tawsifurrahman/covid19-radiography-database.
